# Construction of novel repeat proteins with rigid and predictable structures using a shared helix method

**DOI:** 10.1038/s41598-017-02803-z

**Published:** 2017-06-01

**Authors:** Suk-Jun Youn, Na-Young Kwon, Ji Hyun Lee, Jin Hong Kim, Jinwoo Choi, Hayyoung Lee, Jie-Oh Lee

**Affiliations:** 1 Department of Chemistry, Korea Advanced Institute of Science and Technology, Daejeon, 34141 Korea; 2 Institute of Biotechnology, Chungnam National University, Daejeon, Korea

## Abstract

Generating artificial protein assemblies with complex shapes requires a method for connecting protein components with stable and predictable structures. Currently available methods for creating rigid protein assemblies rely on either complicated calculations or extensive trial and error. We describe a simple and efficient method for connecting two proteins via a fused alpha helix that is formed by joining two preexisting helices into a single extended helix. Because the end-to-end ligation of helices does not guarantee the formation of a continuous helix, we superimposed 1–2 turns of pairs of connecting helices by using a molecular graphics program. Then, we chose amino acids from the two natural sequences that would stabilize the connecting helix. This “shared helix method” is highly efficient. All the designed proteins that could be produced in *Escherichia coli* were readily crystallized and had the expected fusion structures. To prove the usefulness of this method, we produced two novel repeat proteins by assembling several copies of natural or artificial proteins with alpha helices at both termini. Their crystal structures demonstrated the successful assembly of the repeating units with the intended curved shapes. We propose that this method could dramatically expand the available repertoire of natural repeat proteins.

## Introduction

Repeat proteins, such as ankyrin, armadillo, tricopeptide and leucine-rich repeat (LRR) proteins, have been successfully used in biological drug design and protein engineering^1^. A large library of artificial ankyrin repeats has been generated by mutating solvent-exposed residues in concave protein surfaces^2^. Using this library, clones that bind to target proteins have been selected by ribosome or phage display methods^3^. One clone that can bind to and antagonize vascular endothelial growth factor (VEGF) is currently in phase 3 clinical trials against wet macular degeneration^4^. Artificial LRRs that bind with high affinity to clinically important proteins have been isolated by screening libraries of artificial LRR proteins as well^5, 6^. For example, an LRR protein selected for binding to VEGF effectively suppressed choroidal neovascularization and vascular leakage in a mouse model system and is currently being developed as a therapeutic agent^7^. The curved shape and curvature of the repeat proteins are important for increasing the interaction surface of the target proteins because most of them bind to the concave surface of the repeat proteins.

Each repeat protein family has a characteristic value for its curvature that exhibits little variation. Therefore, the curvature that can be achieved using natural repeat proteins is restricted within a small range. To expand the limited repertoire of natural repeat proteins, those without significant sequence homology to any natural repeat proteins have been produced by a computer-aided method^8^. To design the repeating units, short peptide sequences that can form alpha helices and dock to each other were systematically optimized. The docked helical segments were later connected by short peptide linkers. This method successfully generated 43 repeat proteins with the expected shapes in 83 attempts, as observed via x-ray solution scattering experiments. Furthermore, the structures of 15 of these proteins were determined by x-ray crystallography and were superimposable on the intended structures. Using a related computational method, Doyle *et al*. produced alpha helical repeat proteins with closed, toroid-like architectures^9^. However, the repeat proteins generated by these computational methods are currently limited to simple alpha helical structures.

Here, we propose a method for generating proteins and protein complexes with complicated shapes using fusions of alpha helices. To design amino acid sequences that can stabilize the alpha helical structure of the fusion sites, we superimposed one or two turns of terminal alpha helices of connecting proteins using a molecular graphics program. Then, the amino acid residues important for forming a fused helical structure were chosen from the two natural sequences in the superimposed region. Our method does not require complicated computer calculations for the design process and can be used for the assembly of artificial protein complexes with predictable and rigid structures. We used this method to generate artificial repeat proteins with curved shapes. As the model system, we chose a domain of protein A as the repeating module and produced repeat proteins by assembling these modules. Successful helix fusion was confirmed by determining the crystal structures of the designed repeat proteins. We also succeeded in producing novel repeat proteins that have a protein A-designed helical repeat 14 (DHR14) fusion protein as the repeating unit. Our shared helix method is simple, efficient and could have broad applications not only for the design of repeat proteins but also in protein crystallization, cryo-electron microscopy (cryo-EM) analysis and protein engineering.

## Results

### Application of the shared helix method to the fusion of diverse protein modules

**Figure 1:**
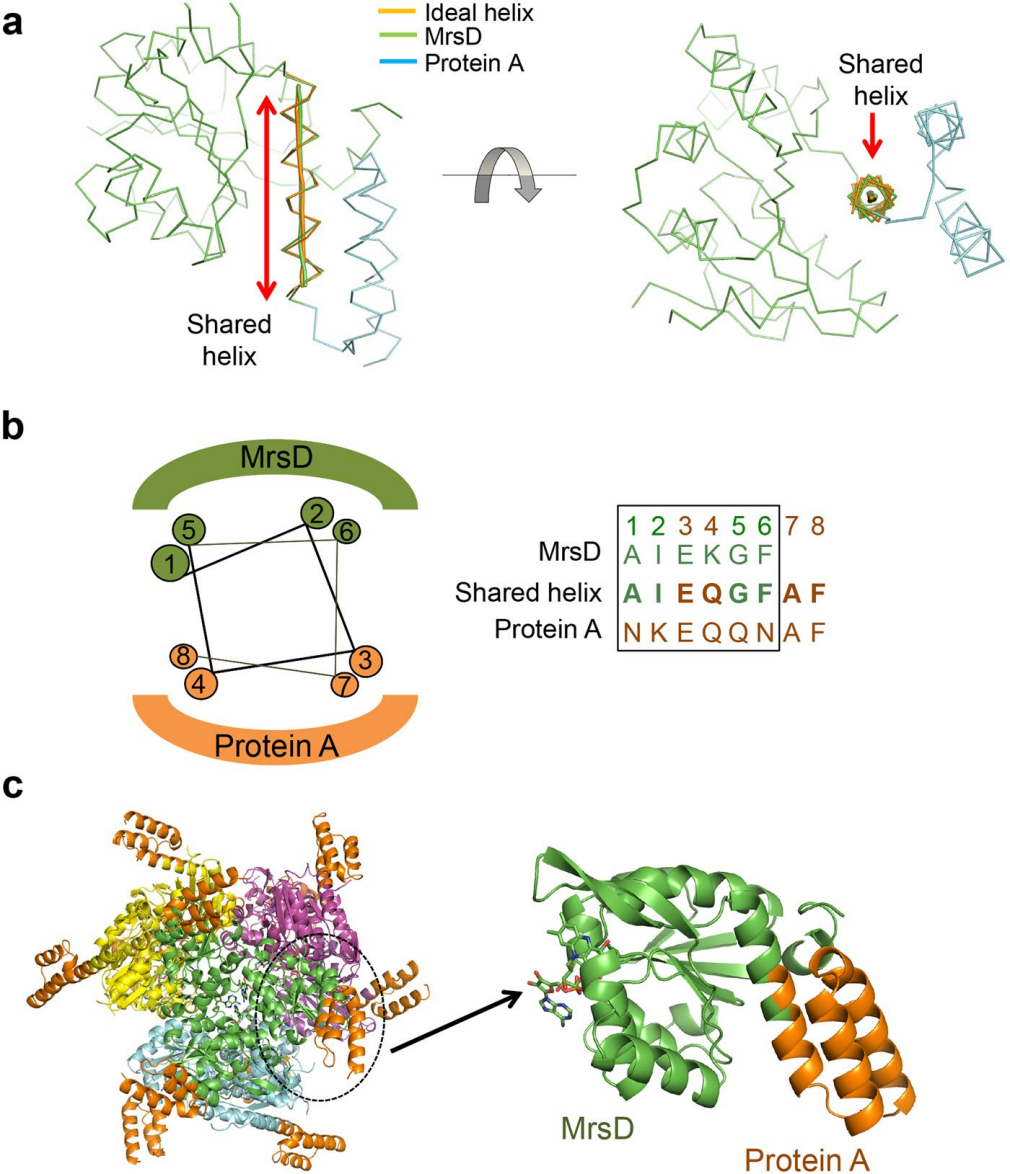
Fusion of MrsD and protein A by the shared helix method. (**a**) The helix docking strategy. An ideal helix from the 1RHG structure of PDB database is first docked to the C-terminal helix of MrsD. Then, the N-terminal helix of protein A is docked to the ideal helix while avoiding steric collision between the two proteins. See “Methods” for details. (**b**) The amino acid selection strategy. Amino acids of MrsD are selected for positions 1, 2, 5 and 6, whose side chains point to the hydrophobic core of MrsD. Similar residues of protein A are selected for positions 3, 4, 7 and 8. (**c**) Crystal structure of the MrsD-protein A fusion protein. A magnified view of the structure of one of the twelve subunits is shown on the right. The structure of a FAD molecule bound to MrsD is drawn as sticks.

To connect two proteins using the shared helix method, we first defined a model alpha helix whose bending and twist angles are close to the average values of naturally occurring alpha helices. The ideal helix that we chose was extracted from the 1RHG structure, amino acids 143–172, of the Protein Data Bank (PDB) database^10^. The twist and bending angles of the chosen alpha helix were 99.2 and 7.4 degrees, respectively^11^. To design the shared helix, the ideal helix was structurally aligned with the C-terminal helix of protein 1 and the N-terminal helix of protein 2, as shown in Fig. 1a, while superimposing the two helices by 1–3 helical turns using a molecular graphics program. In the superimposed region, amino acids were selected from one or the other of the two natural sequences in such a way as to stabilize the shared helix. That is, protein 1 residues were chosen for amino acid positions pointing to the core of protein 1, and protein 2 residues were chosen for positions those pointing to the core of protein 2 (Fig. 1b).

We selected several model proteins to test the efficacy of the shared helix method. First, we chose proteins with terminal alpha helices. Second, all the selected proteins were easily produced in *E. coli* and readily crystallizable. Third, we selected proteins with diverse structures. The first example we chose was the mersacidin decarboxylase (MrsD) cage protein and a protein A domain. MrsD is a homo-oligomeric flavin-containing Cys decarboxylase (HFCD) flavoprotein involved in the biosynthesis of the lantibiotic mersacidin. It forms a large cage-like structure composed of 12 subunits^12^. Protein A is a surface protein of *Staphylococcus aureus* that modulates host immune responses by binding to the constant region of immunoglobulins^13^. The C-terminus of MrsD has an alpha helix of 13 amino acid residues. We superimposed 6 amino acids of this helix with the N-terminal helix of the protein A domain (Fig. 1b and Supplementary Table 1). In the superimposed region, MrsD residues were chosen for amino acid positions pointing to the core of MrsD, and residues of protein A were chosen for those pointing to the core of the protein A. The MrsD-protein A fusion protein designed in this way was successfully produced in *E. coli* as a soluble protein that was resistant to subtilisin digestion and crystallizable. In the crystal structure, the MrsD and protein A modules are connected by a long fused alpha helix (Fig. 1c). The bending and twist angles of the fused helix are 5.5 and 98.8 degrees, respectively, closely resembling those of the ideal helix used in the design (Supplementary Fig. 1). The helical angles were calculated using the HELANAL web server^14^.

**Figure 2:**
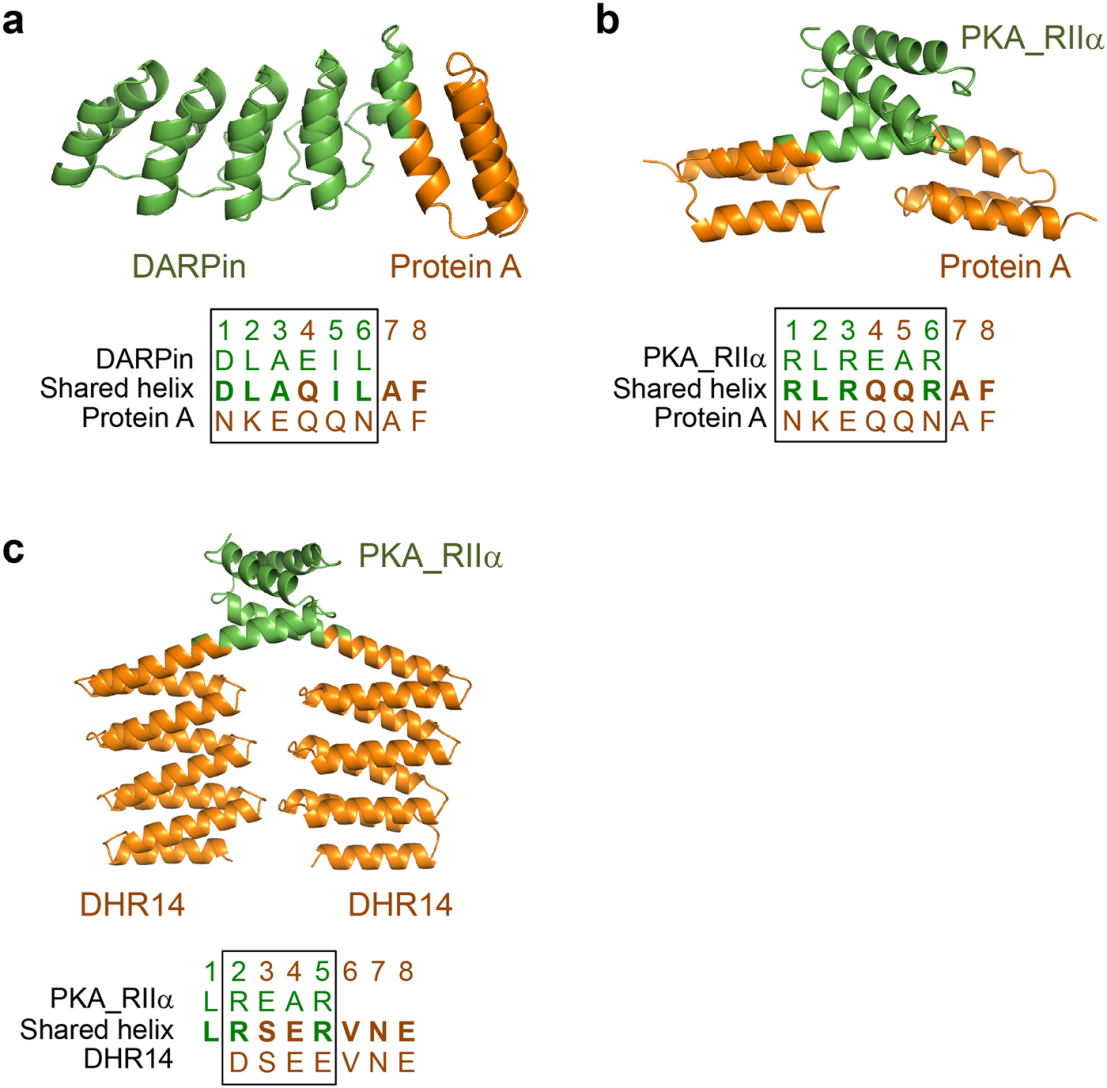
Fusions of DARPin-protein A, PKA_RIIα-protein A and PKA_RIIα-DHR14 by the shared helix method. (**a**) Crystal structure of the DARPin-protein A fusion protein. The amino acid sequences of the fusion helix area of DARPin, the fusion protein and protein A are aligned and shown below. The shared area of the fusion helix is marked with a box. (**b**) Crystal structure of the PKA_RIIα (dimerization domain of the regulatory subunit of protein kinase A)–protein A fusion protein. (**c**) Crystal structure of the fusion protein of PKA_RIIα and DHR14.

The second example we chose was a fusion of an artificial ankyrin protein (designed ankyrin repeat protein (DARPin)) and the protein A domain^3^ (Fig. 2a). The DARPin protein, mbp3-16, had been previously selected by ribosome display from a large library of mutant ankyrin repeat proteins as a protein that binds to maltose-binding protein (MBP)^15^. The fusion helix was formed by superimposing 6 amino acids of the C- and N-terminal helices of the ankyrin and protein A domains, respectively, as described previously (Fig. 2a and Supplementary Fig. 2). The crystal structure shows that the two helices were fused into a long continuous helix with nearly ideal helical geometry (Supplementary Fig. 3)

The third example we chose was a fusion between the protein kinase A regulatory motif (PKA_RIIα) and the protein A module (Fig. 2b). PKA_RIIα is composed of two alpha helices and forms a stable homodimer^16^. For the helix design, 6 amino acids of the PKA module were superimposed with the N-terminal helix of protein A (Fig. 2b and Supplementary Fig. 4). As expected, protein A was connected to the PKA module by a continuous fused helix in the crystal structure (Supplementary Fig. 5). Because the PKA module forms a stable dimer, this method can be used to generate forced homodimeric proteins that may be useful in designing artificial protein nanostructures. As a control, we produced a fusion protein of PKA_RIIα and protein A by the end-to-end fusion of the connecting helices (Supplementary Table 2). The resulting protein was readily produced in *E. coli* and could be crystallized. The crystal structure shows that the PKA_RIIα-protein A (end-to-end) fusion protein shows substantial structural variation at the fusion site. Asymmetric unit of the crystal has four fusion proteins. Structure of one of them is disordered at the fusion site and the protein A domain is not visible in the electron density map. The remaining three fusion proteins in the crystal show substantial structural variation at the fusion sites and their protein A domains could not be superimposed (Supplementary Fig. 6). In comparison, the fusion proteins PKA_RIIα-protein A, MrsD-protein A and DARPin-protein A ligated by the shared helix method have negligible structural flexibility at the fusion sites.

In the fourth test case, we connected PKA_RIIα to a synthetic repeat protein, DHR14 (Fig. 2c). The latter was previously designed by a computational docking method, and its structure was confirmed by x-ray crystallography^8^. The helix fusion was designed by superimposing four amino acids of the second helix of the PKA_RIIα and the first alpha helix of DHR14 (Fig. 2c and Supplementary Fig. 7). In the crystal structure, the two helices were fused into a single extended helix, as expected (Supplementary Fig. 8). The DHR14 module appears to be rotated ~8 degrees relative to the initial design, presumably because of an opportunistic interaction between the loops connecting the third and fourth repeats of the two DHR14 structures (Supplementary Fig. 8d,e). As a control, we also produced a fusion protein of PKA_RIIα and DHR14 by the end-to-end fusion of the connecting helices (Supplementary Table 2). The fusion helices are directly ligated without any intervening amino acids. The resulting proteins were produced in *E. coli* but could not be crystallized under our standard set of 1,152 screening conditions. This is presumed due to high structural flexibility at the fusion site because isolated PKA_RIIα and DHR14 proteins could be crystallized under multiple conditions using the same screening solutions.

### Construction of a large symmetric protein binder for single-particle cryo-EM analysis

**Figure 3:**
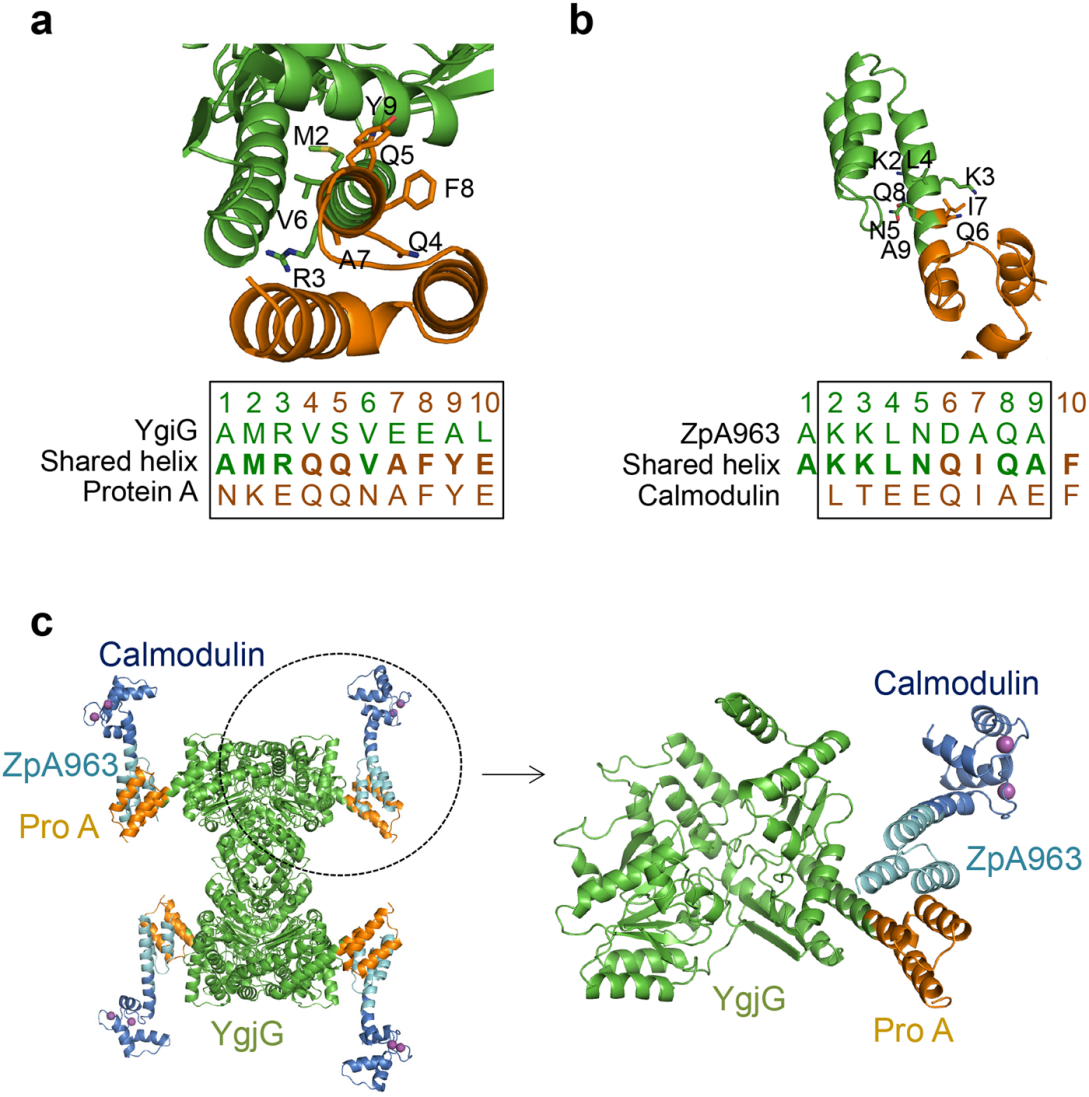
Construction of a tetrameric protein complex by the shared helix method. Fusion strategies of the YgiG-protein A (**a**) and the ZpA963-calmodulin (**b**) by the shared helix method. Models of the shared helix regions of the fusion proteins are shown above. The amino acid sequences of the shared fusion helix are written below. Sequences of the shared region are marked with a box. (**c**) The crystal structure of the YgjG-protein A and the Zpa963-calmodulin complex. A magnified view of one of the four subunits is shown on the right. The calcium ions bound to the calmodulin domain are drawn as purple balls.

To produce protein complexes useful for cryo-EM analysis, we designed a large tetrameric protein complex. It is believed that proteins should be rigid and larger than ~100 kDa for high-resolution cryo-EM analysis^17^. Thus, a protein binder that is large and symmetric is needed to increase the effective size of smaller target proteins. The *E. coli* putrescine aminotransferase, YgjG, was chosen for this purpose. The crystal structure of YgjG shows that it forms a homotetramer, with each monomeric subunit containing 460 amino acids^18^. The protein A-ZpA963 heterodimer was chosen as the linker connecting YgjG and the target protein. ZpA963 is a variant of protein A that non-covalently binds to protein A with high affinity^19^. As a test case, we used a calmodulin domain with molecular weight of ~10 kDa as the target protein. For protein assembly, the N-terminal helix of the protein A domain was connected to the C-terminal helix of YgjG by superimposing ten amino acids (Fig. 3a and Supplementary Fig. 9a,b). ZpA963 and the calmodulin domain were also connected by a helix fusion by superimposing eight amino acids (Fig. 3b and Supplementary Fig. 9c,d). The overall size of the hetero-octameric YgjG-protein A-ZpA963-calmodulin complex is 268 kDa, which is large enough for high-resolution cryo-EM. The crystal structure and a binding assay using gel-filtration chromatography revealed that the complex forms a rigid hetero-octameric complex, as intended (Fig. 3c and Supplementary Fig. 10).

**Figure 4:**
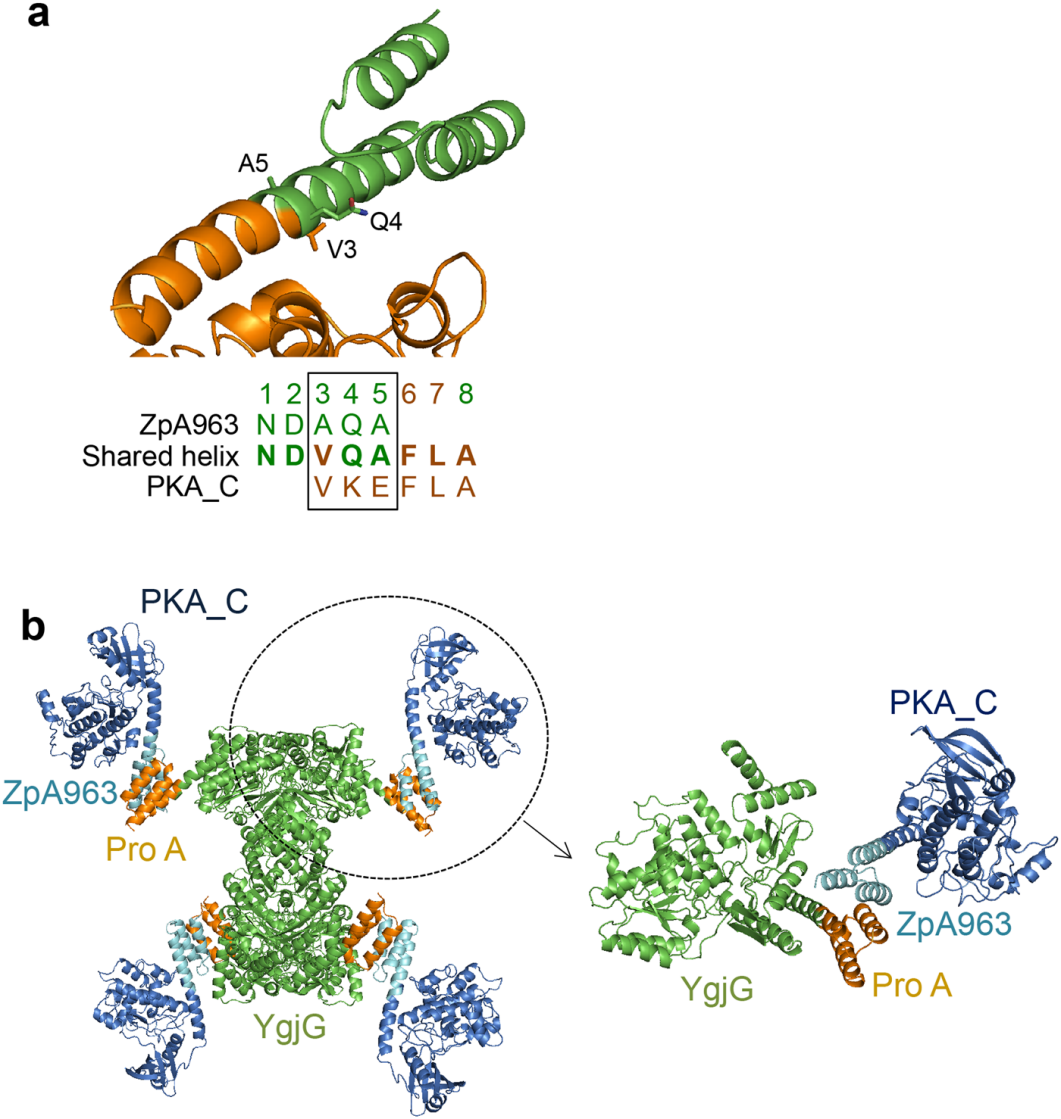
Construction of a YgjG-protein A-ZpA963-protein kinase A complex by the shared helix method. (**a**) Fusion strategy of ZpA963 and PKA_C, the catalytic domain of protein kinase A, by the shared helix method. A model of the shared helix region of the fusion protein is shown above. The side chains of the amino acids in the shared region are drawn as sticks. The amino acid sequences of the fusion helix region are written below. The sequences of the shared region are marked with a box. (**b**) The crystal structure of the YgjG-protein A and the ZpA963-PKA_C complex. A magnified view of one of the four subunits is shown on the right.

To demonstrate that target proteins other than calmodulin can be attached to the YgjG backbone with a rigid and predictable structure, we merged the C-terminal helix of ZpA963 to the N-terminal helix of the catalytic domain of PKA (PKA_C) by the shared helix method (Fig. 4 and Supplementary Fig. 11). The fusion helix was designed by superimposing the last three amino acids of the C-terminal helix of ZpA963 with the first turn of the N-terminal helix of PKA_C using a molecular graphics program (Fig. 4a). The resulting fusion protein could be produced in *E. coli* and readily crystallized. Binding analysis using gel-filtration chromatography revealed that the fusion proteins formed a stable complex, as intended (Supplementary Fig. 12). The crystal structure demonstrates that the two helices are successfully fused and become a single extended helix without altering the structure of the PKA catalytic domain (Fig. 4b). As a control, we also produced a fusion protein of the YgjG and protein A by the end-to-end fusion of the connecting helices (Supplementary Table 2). The resulting protein was produced in *E. coli* and mixed with ZpA963-calmodulin and ZpA963-PKA_C fusion proteins, respectively, generated by the shared helix method (Figs 3b and 4a). The purified YgjG-protein A (end-to-end) protein formed stable complexes with these two ZpA963 fusion proteins but could not be crystallized, presumably because of high structural flexibility at the fusion site.

### Design of an artificial repeat protein using a protein A domain as the repeat unit

**Figure 5:**
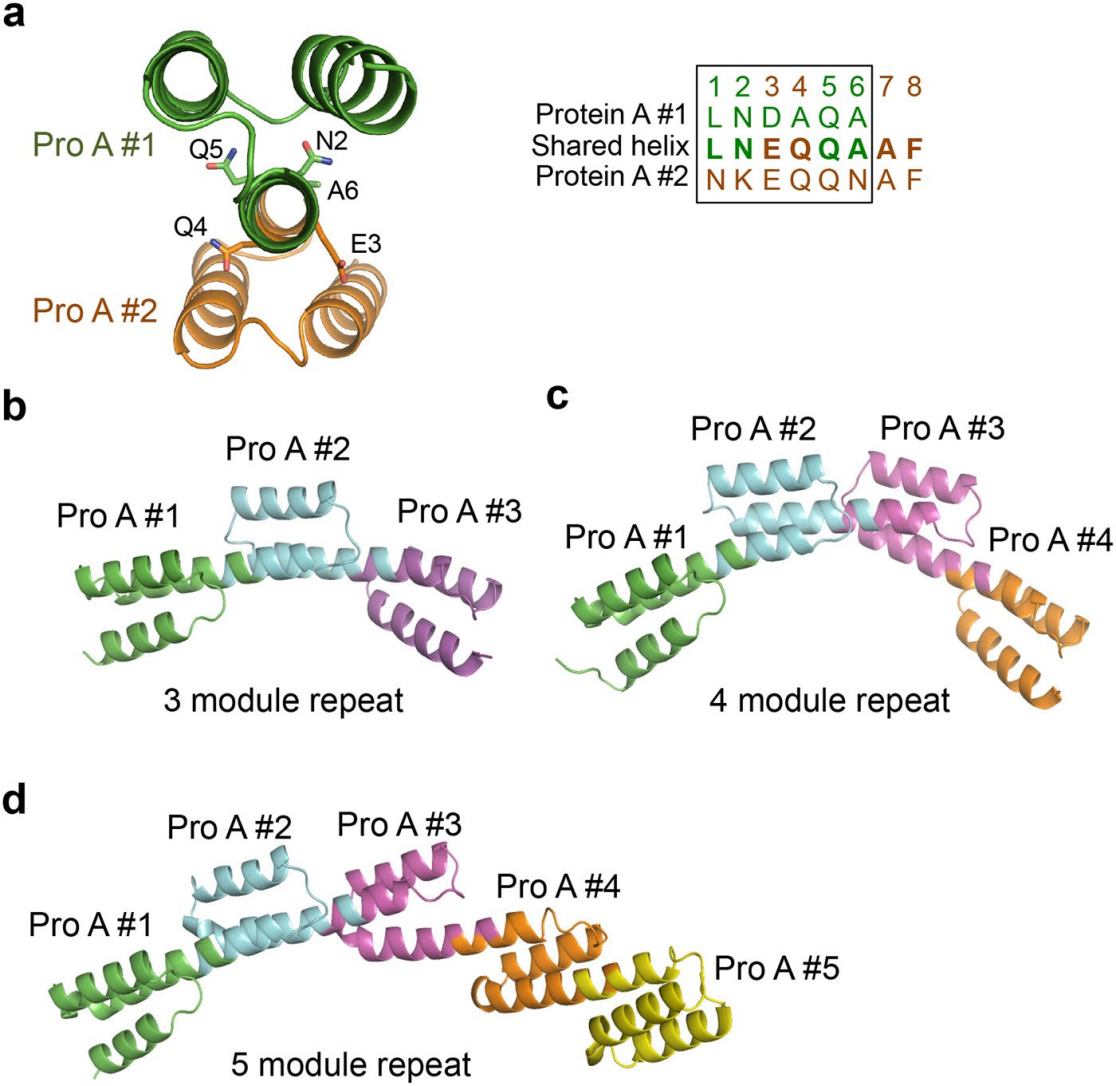
Construction of a repeat protein using the protein A domain as the repeat module. (**a**) Fusion strategy of protein A domains by the shared helix method. The amino acid sequences of the shared helix are shown on the right. The side chains of amino acids in the shared region are drawn as sticks. (**b**) The crystal structure of a repeat protein with three repeat modules. (**c**) The crystal structure of a repeat protein with four repeat modules. (**d**) The crystal structure of a repeat protein with five repeat modules.

We thought that proteins with alpha helices at both their N- and C-termini might be fused to generate artificial repeat proteins by connecting the C-terminal helix of the repeating unit to the N-terminal helix of the next repeating unit. To test this design strategy, we used a protein A domain as the repeating module and generated 3-, 4- and 5-module repeat proteins (Fig. 5a and Supplementary Fig. 13). All these repeat proteins were successfully produced in *E. coli* and could be crystallized. In the crystal structures, all the repeating units were connected as designed (Fig. 5b–d). The twist and bending angles of the fused helices were very similar to those of the ideal helix. Interestingly, the protein A modules in the crystal structures, especially the last ones, exhibited significant structural variation (Supplementary Fig. 14a), and close structural analysis demonstrated that this structural diversity was due not to the helix fusion but to the intrinsic flexibility of the protein A module. For this analysis, we downloaded many of the protein A structures deposited in the PDB database and aligned them structurally. The analysis showed that the three helices also varied substantially in orientation and position in wild-type protein A structures (Supplementary Fig. 14b); this result implies that crystal packing selects one specific conformation of protein A from a mixture of several available structures.

### Design of an artificial repeat protein with a mixed repeat unit

**Figure 6:**
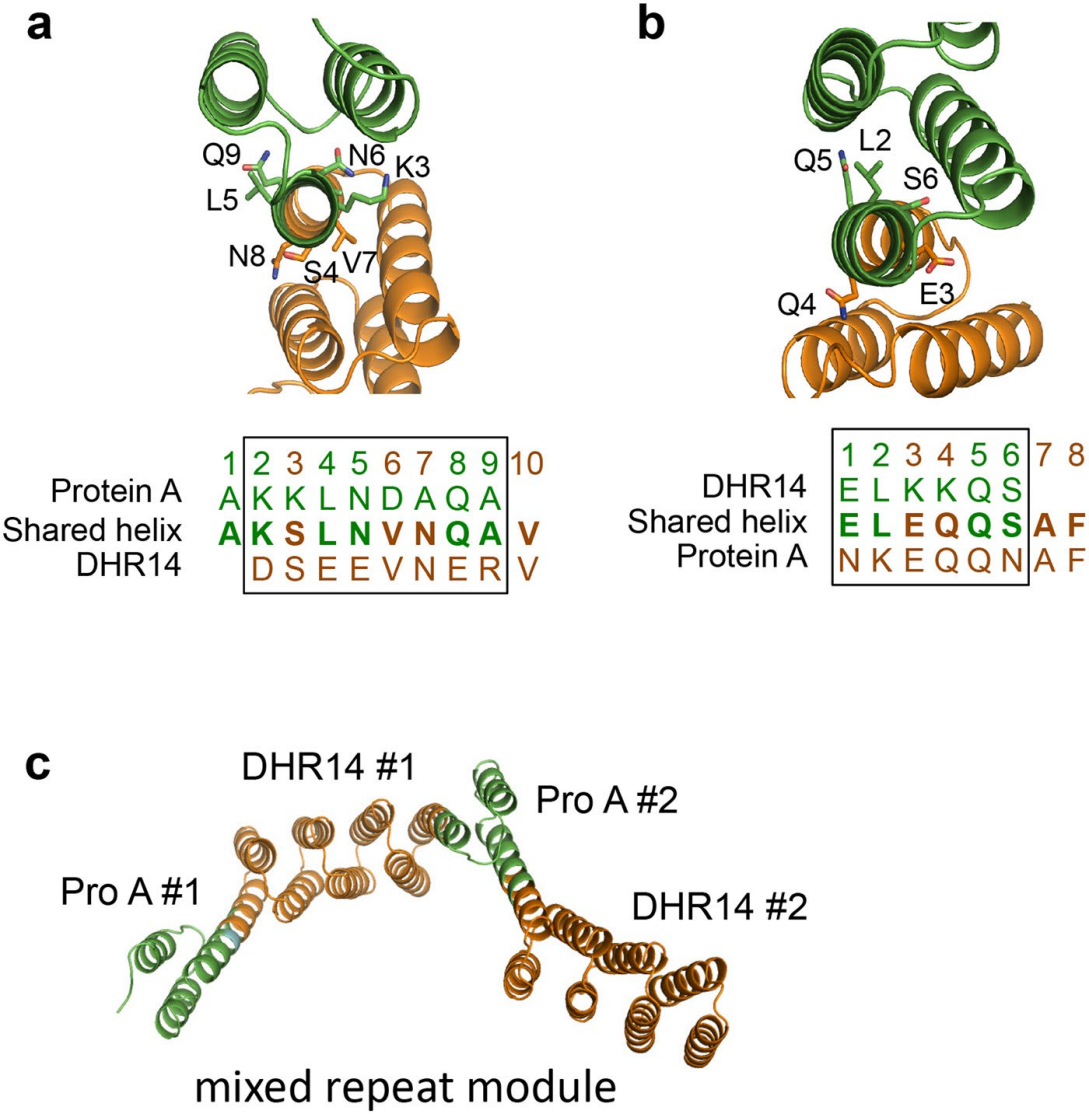
Construction of a repeat protein using the protein A-DHR14 fusion as the mixed repeat module. Fusion strategies of the (**a**) protein A-DHR14 and (**b**) DHR14-protein A fusions by the shared helix method. Models of the shared helix regions of the fusion protein are shown above. The side chains of the amino acids in the shared region are drawn as sticks. The amino acid sequences of the fusion sites are shown below. The shared regions are marked with boxes. (**c**) The crystal structure of a repeat protein composed of two protein A-DHR14 repeat modules.

We designed another repeat protein using a protein A-DHR14 fusion protein as the repeating unit. DHR14 has four modules containing two consecutive helices and a short connecting loop^8^. The repeating unit was generated by fusing protein A and DHR14 by superimposing 8 amino acids using the shared helix method (Fig. 6a and Supplementary Fig. 15). Then, the repeat modules were connected by superimposing 6 amino acids to form the repeat protein (Fig. 6b and Supplementary Fig. 16). The crystal structure again showed that the two helices had been fused into merged and extended helices with a geometry closely matching that of an ideal helix (Fig. 6c).

## Discussion

In this report, we demonstrated that the shared helix method can be used to connect a variety of protein components and generate useful protein structures. As described in the Introduction, computational methods are important tools for developing new repeat proteins^8, 9^. However, the repeat modules that can be designed by computational methods are currently limited to simple alpha helical peptides. Here, we show that the shared helix strategy can generate a large number of novel repeat proteins with longer and more complex repeating modules, without the need for complicated calculations.

We and others have demonstrated that the simple end-to-end ligation of helices does not create a single continuous helix, and therefore, the structures of the resulting fusion proteins cannot be predicted with high accuracy. In the present research, we have shown that the PKA_RIIα-DHR14(end-to-end) fusion and the YgiG-protein A(end-to-end) fusion in complex with two different ZpA963 fusion proteins could not be crystallized, presumably because of high structural flexibility at the fusion sites. The PKA_RIIα-protein A(end-to-end) fusion could be crystallized but the connecting helices in the crystal have substantial structural variation at the fusion sites. Therefore, overall structure of the fusion protein cannot be predicted with high confidence. These findings demonstrate that the shared helix method is useful for merging alpha helices into a single extended helix with a rigid and accurately predictable structure.

Previously, we fused protein A to the calmodulin N-terminal domain by the end-to-end helix fusion method with a short linker containing three alanines^20^. Alanines were chosen as the linker because it is small and ranks top in several helix propensity scales^21^. Among the four fusion proteins designed, only one could be crystallized. The remaining three proteins could be produced but not crystallized. In the structure of the crystallized fusion protein, the two helices fail to merge into one extended helix. Instead, the helical turn at the fusion site is unwound and becomes an irregular loop (Supplementary Fig. 17a). The fusion protein could be crystallized because the relative structure of the fusion partners is stabilized by an opportunistic disulfide bridge between the two proteins. We also generated seventeen fusion proteins by connecting the C-terminal helix of an artificial ankyrin repeat protein and the N-terminal alpha helix of protein A by the end-to-end fusion method. Only one of these fusion proteins could be crystallized. The crystallization of the remaining sixteen fusion proteins failed presumably because the alpha helices failed to merge into a single extended helix and because the structures of the fusion sites are too flexible for crystallization. In the crystal structure of the one fusion protein that could be crystallized, the connecting helix is bent significantly at the fusion site. A chemical crosslinker had to be attached to the fusion helix to force it to adopt the structure of an ideal alpha helix.

Other research groups have reported similar results. MBP has been frequently used as a fusion partner for the crystallization of challenging proteins. Because it ends in a C-terminal helix, the end-to-end helix fusion method has been attempted by several research groups^22^. Yang *et al*. connected the C-terminal helix of MBP to a long alpha helix of tetherin^23^. They also used a short linker containing two alanines. The crystal structure revealed that the fusion site becomes an irregular loop instead of forming an extended, fused alpha helix (Supplementary Fig. 17b). Similarly, Jin *et al*. connected the C-terminal helix of MBP to the N-terminal helices of several death domain proteins including human MNDA using a short alanine linker^24^ (Supplementary Fig. 17c–e). They found that the fusion helices become significantly bent and distorted relative to those of an ideal alpha helix. Ke and Wolberger connected the N-terminal helix of the mating-type protein a1 (MATa1) homeodomain to the C-terminal helix of MBP to improve the crystallization conditions of the MATa1 homeodomain^25^. The crystal structure revealed that the two alpha helices are not merged into a single helix (Supplementary Fig. 17f). Instead, the fusion site of the two helices becomes an irregular loop. Furthermore, the linker sequence adopts a different loop structure in another crystal form, suggesting that the linker does not have a stable structure and that the crystal packing interaction can change the structure of the linker. Padilla *et al*. designed several proteins by the end-to-end fusion of alpha helices^26^. The resulting fusion proteins form heterogeneous aggregates, presumably because of the structural flexibility and heterogeneity of the fusion helix. Only after sequence optimization of the fusion site by the trial-and-error method were they able to produce the intended protein cage with a homogeneous structure^27^. Please note that only those fusion proteins with structures that are homogeneous and rigid enough for crystallization were reported in the literature. We suspect that, in the vast majority of cases, fusion proteins connected by the end-to-end helix fusion method were not reported because the high structural flexibility at the fusion site prevents them from crystallizing.

In principle, any proteins that have alpha helices at both the N- and C-termini can be used as repeating modules in the shared helix method. A survey of entries in the current PDB database using the STRIDE program showed that 11,429 of the 119,137 entries have helices at both ends^28^. Of these, we manually inspected 120 entries at random using the following three criteria. First, we ruled out those proteins whose terminal helices were not sufficiently exposed to form a helix fusion. Second, we excluded proteins that form multimeric complexes, and, finally, we eliminated proteins in which the two terminal helices were too close together to be used as repeat units because there was no space for simultaneous helix fusion. Based on these criteria, 26 of the 120 entries were deemed suitable as candidate repeating units. If this proportion was similar among all the 11,429 PDB entries with two terminal helices, there would be ~2,500 proteins that could be used as repeating units. In the current PDB database, approximately 37% are estimated to be non-redundant structures (see http://www.rcsb.org/pdb/statistics/clusterStatistics.do). Therefore, ~900 of the ~2,500 candidates might be non-redundant and have structures suitable for use as repeating modules. By connecting these proteins as repeating units or combining a few as mixed repeating units, as shown in Figs 5 and 6, we would be able to generate a virtually unlimited number of novel repeat proteins by the shared helix method. Some of these could be useful in drug design or other biotechnological applications.

The overall shapes of the two repeat proteins that we produced resemble the horseshoe-like shapes of natural repeat proteins (Supplementary Fig. 18). In the repeat proteins, the concave surface is frequently used to bind other proteins presumably because that shape maximizes the interaction surface. The curvature of the horseshoe-like structure is thus critical for the function of repeat proteins. Each repeat protein family has a characteristic value for its curvature, which exhibits little variation. We have shown above that we could generate repeat proteins with a great variety of curvatures via the shared helix method by employing different repeat modules. This technique will be useful for developing repeat proteins that bind target proteins with unusual shapes.

The shared helix method can be used to crystallize many challenging proteins, including transmembrane proteins and large protein complexes. While we were preparing this manuscript, Batyuk *et al*. reported connecting two proteins—an artificial ankyrin repeat protein, DARPin, and a stabilized *E. coli* lactamase—by a similar helix fusion method^29^. They proposed that the DARPin-lactamase fusion would be useful for crystallizing target proteins associated with DARPin because the lactamase component would provide the protein complex with a new crystallizable surface. In this article, we have shown that the shared helix method is not limited to ankyrin repeats and lactamase but can be generally used to connect a variety of natural and non-natural proteins. Moreover, this method is not limited to protein crystallization but can be expanded to generate a variety of useful artificial proteins, including repeat proteins.

In this research, we used proteins with terminal alpha helices for fusion. However, small guest proteins containing terminal alpha helices can be inserted into loop regions preceding or following an internal alpha helix without disturbing the overall structure of the host proteins, as shown previously by many laboratories. For example, lysozyme or cytochrome b_562_RIL has been successfully inserted into a flexible internal loop to improve the crystallization behavior of many G protein-coupled receptor (GPCR) proteins (Supplementary Fig. 19)^30^. The shared helix method can be applied to fuse two proteins (i.e., the host and the inserted guest proteins) to connect them and achieve predictable and rigid structures. This method could improve the success rate of GPCR crystallization because the fusion proteins connected by the shared helix method will have a higher chance of crystallization than those connected by the end-to-end helix fusion method.

Using the calmodulin and PKA domains as model proteins, we demonstrate that the shared helix strategy can be used to increase the effective size of proteins and to enforce four-fold symmetry. This method could be useful for studying the structures of many proteins that cannot be crystallized for x-ray crystallographic analysis and are too small for cryo-EM analysis. GPCRs and protein kinases are arguably the most important targets of drug discovery. However, even after decades of research, the structures of only 5% of human GPCRs and 40% of human protein kinases have been determined by x-ray crystallography. Additionally, GPCRs and protein kinases are too small for high-resolution cryo-EM analysis. These proteins are suitable targets for our shared helix method because the structures of these proteins can be predicted by homology modeling with reasonable accuracy. Therefore, ZpA963 can be fused to the GPCR or kinases by the shared helix method. The resulting fusion proteins can be attached to the 4-fold symmetric YgjG-protein A backbone, as shown in Figs 3 and 4. The hetero-octameric complexes generated should have sizes large enough for cryo-EM study.

In conclusion, we have developed the shared helix method and demonstrated that it is a simple and efficient way to connect protein components to form stable and predictable structures. Using this method, we have generated artificial repeat proteins and large symmetric protein complexes. Future research will be directed toward the practical application of this method in structural biology and biological drug design.

## Methods

### Cloning and preparation of fusion proteins

Genes encoding the fusion proteins were cloned into expression vectors (Supplementary Tables 1–3). To produce the fusion proteins, the recombinant plasmids were transformed into *E. coli* strain BL21(DE3), and the transformed cells were cultivated at 37 °C in lysogeny broth (LB) medium supplemented with 50 μg/ml kanamycin or ampicillin. At an optical density at 600 nm (O.D. 600) of 0.6, protein production was induced by adding isopropyl β-D-1-thiogalactopyranoside (IPTG) to a final concentration of 0.5 mM. The induced cells were incubated for an additional 20 hours at 17 °C, harvested, resuspended in a lysis buffer (20 mM Tris pH 8.0, 200 mM NaCl, 0.5 mM phenylmethane sulfonyl fluoride (PMSF)) and homogenized using a microfluidizer (Microfluidics, USA). Cell debris was removed by centrifugation, and supernatants were loaded on to a Ni-NTA (Incospharm, Korea) or amylose agarose (New England Biolabs) column. The hexahistidine- or MBP-tagged fusion proteins were eluted with lysis buffer containing either 250 mM imidazole or 10 mM maltose. To remove the purification tags, the eluted proteins were treated overnight with 1% thrombin. The cleaved proteins were further purified by ion exchange and Superdex 200 gel-filtration chromatography, and fractions containing the proteins were collected and concentrated for crystallization. For calmodulin fusion proteins, 5 mM CaCl_2_ was added to all the buffers.

### Molecular modeling of the shared helices

For the design of the shared alpha helix connecting the two proteins, the C-terminal alpha helix of the first protein and the N-terminal alpha helix of the second protein were superimposed using the “LSQ superpose” command of a molecular graphics program, Crystallographic Object-Oriented Toolkit (COOT), as shown in Fig. 1a ^31^. An ideal helix from the 1RHG structure, amino acids 143–172, of the PDB database was used to improve the accuracy of superimposition because the overlapped region of two helices is often too short for reliable superimposition. In these cases, the alpha helices of the connecting proteins are superimposed on the ideal helix structure, and several amino acids are overlapped at the fusion site. The amino acids of the fusion region were selected from the two possible choices for each fusion partner, as described in the “Results” section and shown in Fig. 1b. The conformations of the side chains at the fusion sites were adopted from those of the isolated wild-type structures. Protein models that created severe steric collisions that were unavoidable by using the “Rotamers” command were removed from the fusion candidate list. The “residue environment distances” command of the COOT program lists inter-residue distances and helps find potential steric collisions. Residues that became closer than 2.5 Å were considered to be sterically colliding with each other. Although we used the COOT program for modeling, other molecular viewer programs, including PyMOL and the Swiss PDB viewer, have similar functions and could also be used. Computationally intensive methods, such as energy minimization, molecular dynamic simulation and dead-end elimination, were not attempted.

### Crystallization, data collection and structure determination

Protein crystals were obtained by the vapor diffusion method. The crystallization conditions and cryoprotectants used for data collection are summarized in Supplementary Table 4. The crystals were flash-frozen using liquid nitrogen, and diffraction data were collected at the Pohang Accelerator Laboratory on beam lines 5 C and 7 A. Diffraction images were indexed, integrated and scaled with the HKL2000 program (HKL Research). PHASER was used to perform molecular replacement calculations^32^, and structure solutions were refined and manually corrected using PHENIX, Refmac5 and COOT^31, 33, 34^. The search probes used for the PHASER calculations are summarized in Supplementary Tables 5–7. ProA_4repeats crystals are pseudo-merohedrally twinned with twin fraction 0.11. Therefore, twin law h,-k,-l was applied during the refinement. The data set of ProA_5repeats is severely anisotropic, with diffraction limits of 3.2 Å and 3.0 Å along the a* and c* directions, respectively, but only 3.9 Å along the b* direction. Therefore, to remove the anisotropy and improve the quality of the electron density maps, the Diffraction Anisotropy Server (http://services.mbi.ucla.edu/anisoscale/) was used to perform ellipsoidal truncation and anisotropic scaling^35^. Briefly, the diffraction data were truncated outside the specified ellipsoid of dimensions: 1/3.2 Å, 1/3.9 Å, 1/3.0 Å along a*, b*, and c*, respectively. After elliptical truncation, anisotropic scale factors were applied to reduce the anisotropy. To restore the magnitude of the high-resolution reflections diminished by the anisotropic scaling, an isotropic B factor of −54.7 Å^2^ was applied. These anisotropically scaled data were used for model building and refinement. The crystallographic data are summarized in Supplementary Tables 5–7.

### Data Availability

Atomic coordinates and diffraction data have been deposited in the Protein Data Bank. The code numbers are summarized in Supplementary Tables 5–7.

## Electronic supplementary material

Supplementary Figures and Tables
