# Supplementary material for: Construction of novel repeat proteins with rigid and predictable structures using a shared helix method
Source: Sci Rep. 2017 Jun 1;7:2595. doi: 10.1038/s41598-017-02803-z (PMC5454011; doi:10.1038/s41598-017-02803-z)
Supplement: Supplementary file 1 — Supplementary Figures and Tables [file 41598_2017_2803_MOESM1_ESM.pdf]

Supplementary Information

Construction of novel repeat proteins with rigid and predictable structures using a shared helix method

Suk-Jun Youn*, Na Young Kwon*, Ji Hyun Lee, Jin Hong Kim, Jin Woo Choi, Hayyoung Lee and Jie-Oh Lee

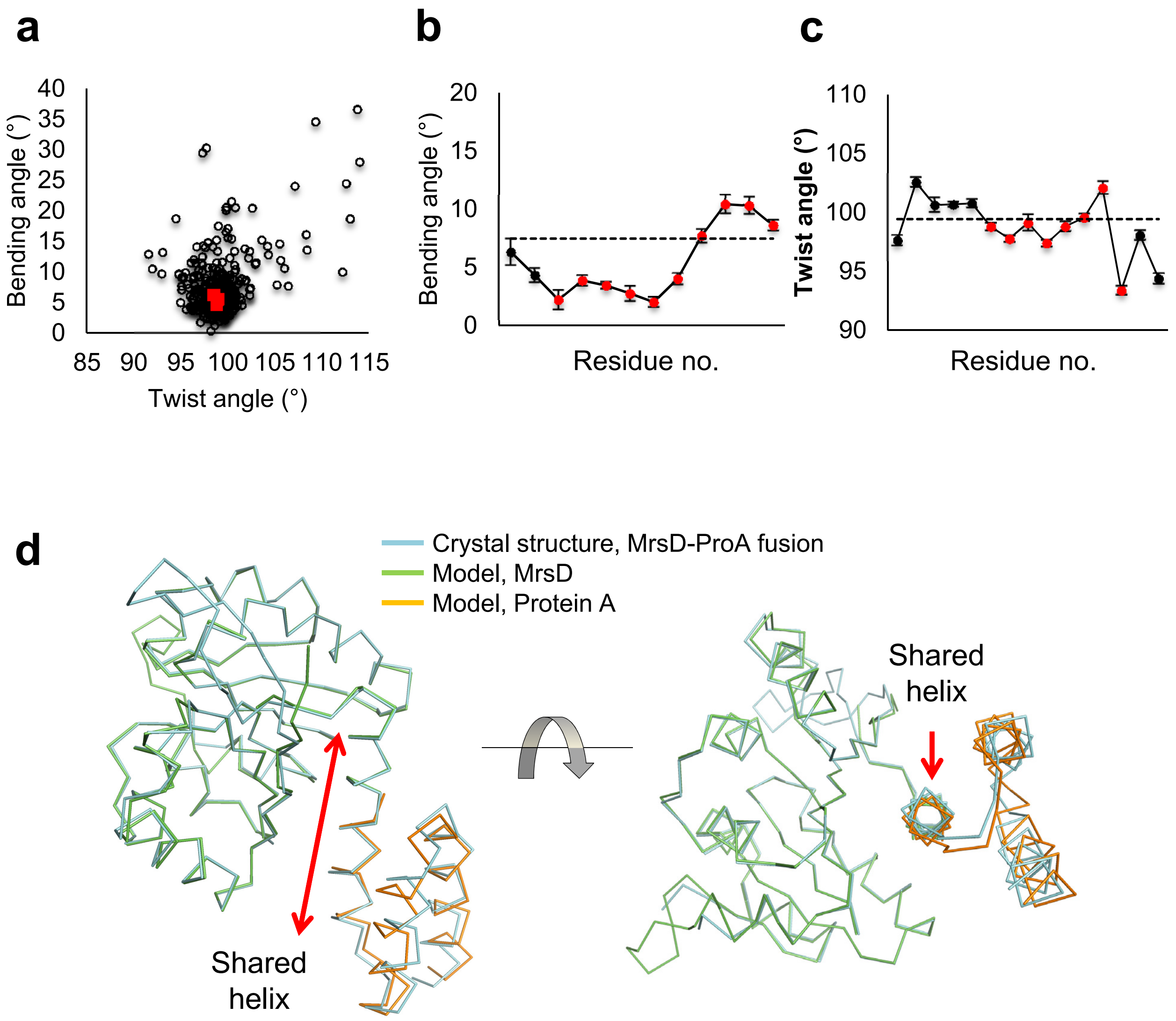

Supplementary Figure 1 | Structural analysis of the connecting helix in the MrsD-protein A fusion protein. (**a**) Plot of the twist and bending angles of 450 alpha helices in 45 standard protein structures [1]. The angles of the connecting helices in the asymmetric unit of the MrsD-protein A crystal are marked with red squares. The crystal contains four fusion proteins in the asymmetric unit. (**b**) Bending angle distribution of the connecting helix. The shared part of the helix is marked with red dots. The bending angle of the ideal alpha helix is drawn as a broken line. (**c**) Twist angle distribution of the connecting helix. The shared part of the helix is marked with red dots. The twist angle of the ideal alpha helix is drawn as a broken line. (**d**) Comparison of the model and crystal structures of the MrsD-protein A fusion. The MrsD parts of the structures are aligned.

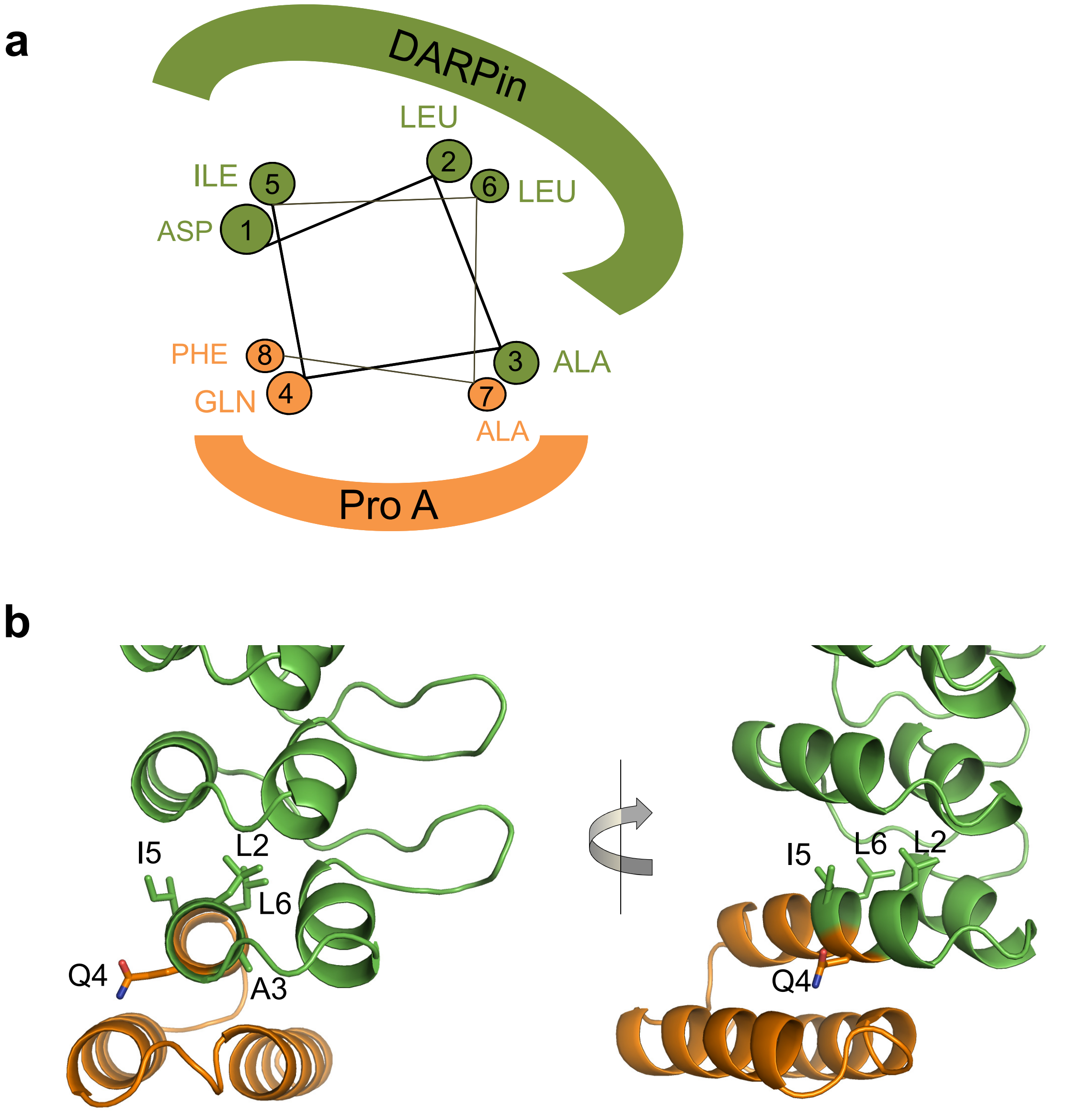

Supplementary Figure 2 | Structure of the connecting helix in the DARPin-protein A fusion. (**a**) Amino acid selection strategy. Amino acids of DARPin are selected for positions 1, 2, 3, 5 and 6 of the shared helix region, and those of protein A are selected for positions 4, 7 and 8 (Fig. 2a). (**b**) The crystal structure of the DARPin-protein A fusion protein. The structure of the connecting helix region is shown. The side chains of the shared region are drawn as sticks.

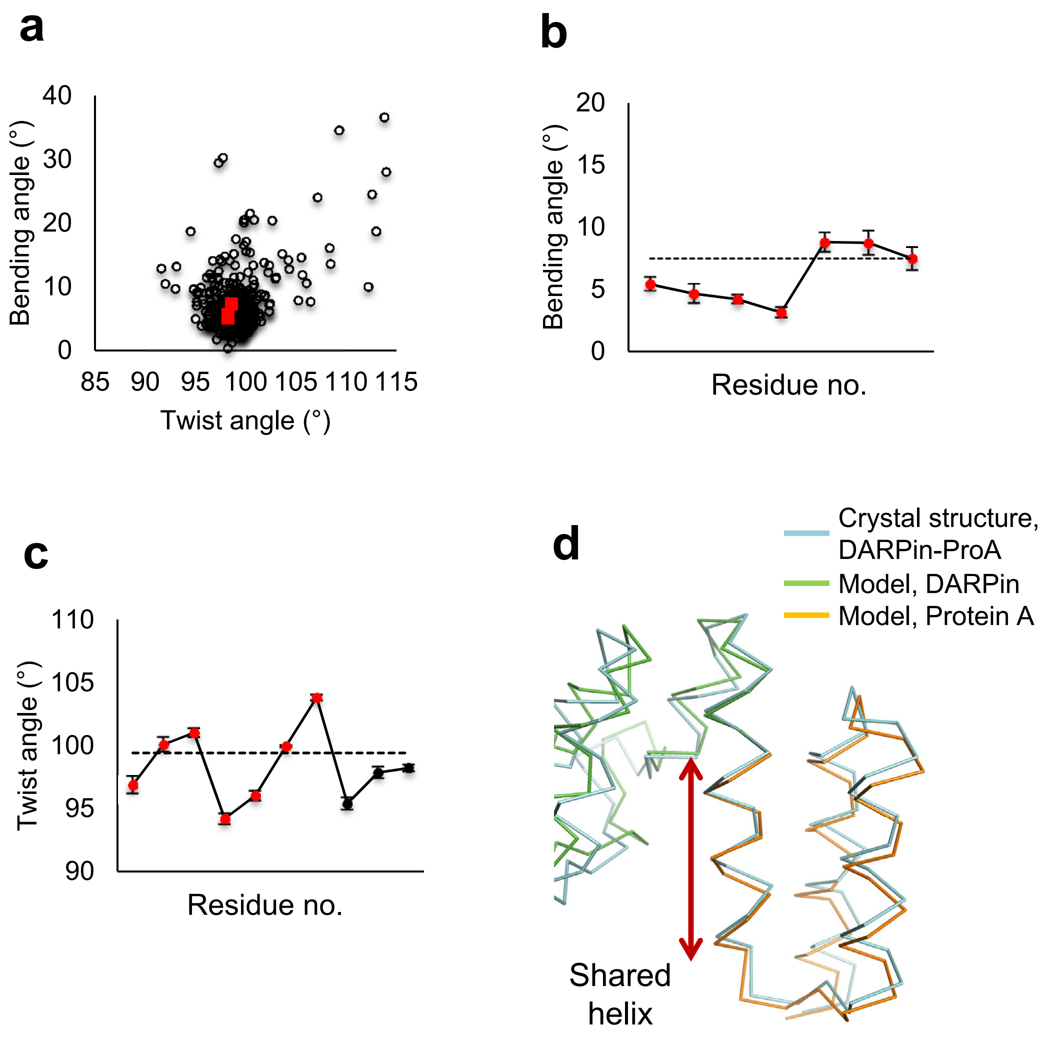

Supplementary Figure 3 | Structural analysis of the connecting helix in the DARPin-protein A fusion protein. (**a**) Plot of twist and bending angles of 450 alpha helices in 45 standard protein structures [1]. The angles of the connecting helices in the asymmetric unit of the DARPin-protein A crystal are marked with red squares. The crystal contains three protein molecules in the asymmetric unit. (**b**) Bending angle distribution of the connecting helix. The shared part of the connecting helix is marked with red dots. The bending angle of the ideal alpha helix is drawn as a broken line. (**c**) Twist angle distribution of the connecting helix. The shared part of the connecting helix is marked with red dots. The twist angle of the ideal alpha helix is drawn as a broken line. (**d**) Comparison of the model and crystal structures of the DARPin-protein A fusion. The DARPin parts of the structures are aligned.

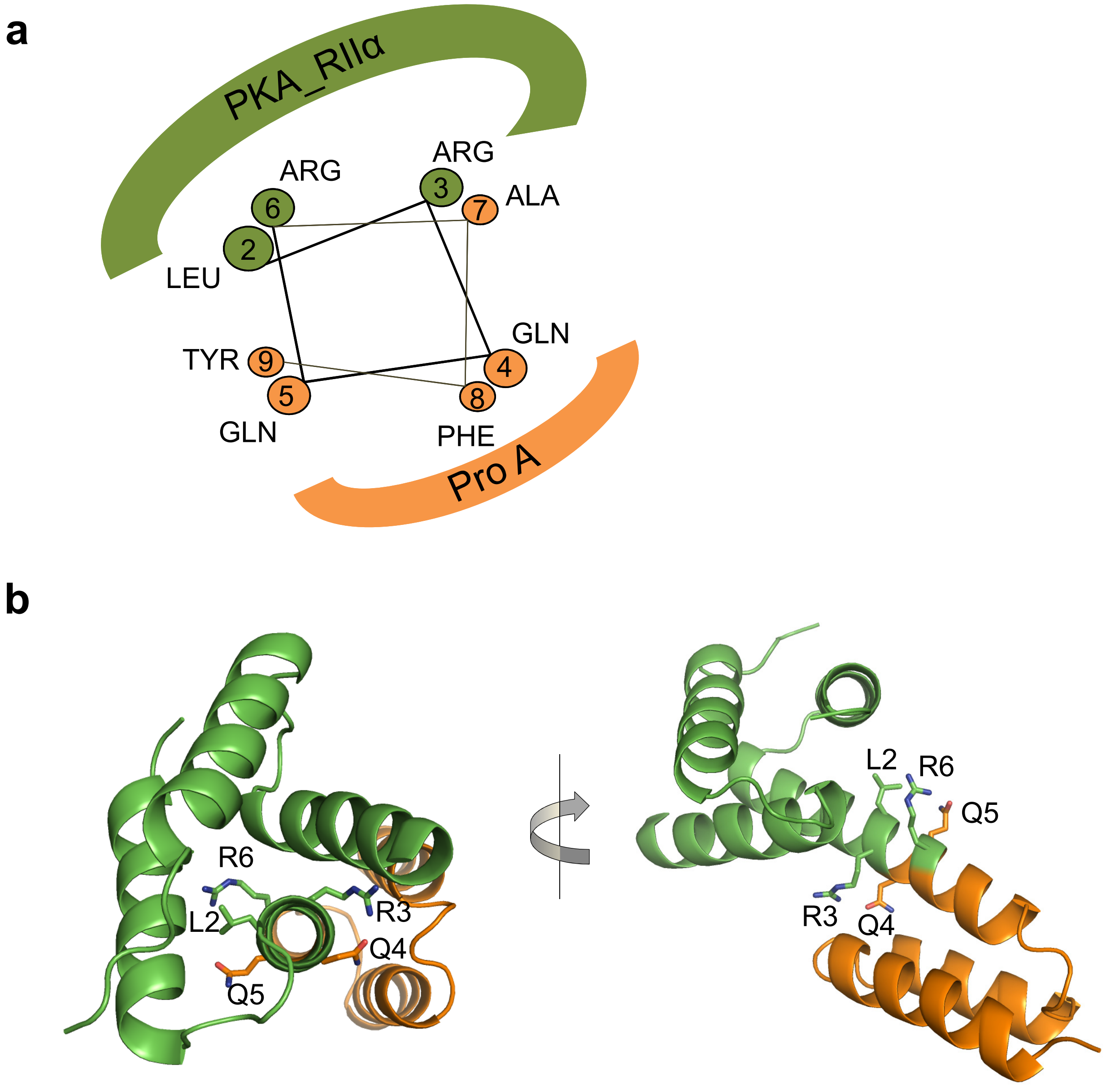

Supplementary Figure 4 | Structure of the connecting helix in the PKA_RIIα-protein A fusion. (**a**) Amino acid selection strategy. Amino acids of PKA_RIIα were selected for positions 1, 2, 3 and 6 of the shared helix region, and those of protein A were selected for positions 4, 5, 7 and 8 (Fig. 2b). (**b**) The crystal structure of the PKA_RIIα-protein A fusion protein. The structure of the connecting helix region is shown. The side chains of the shared region are drawn as sticks.

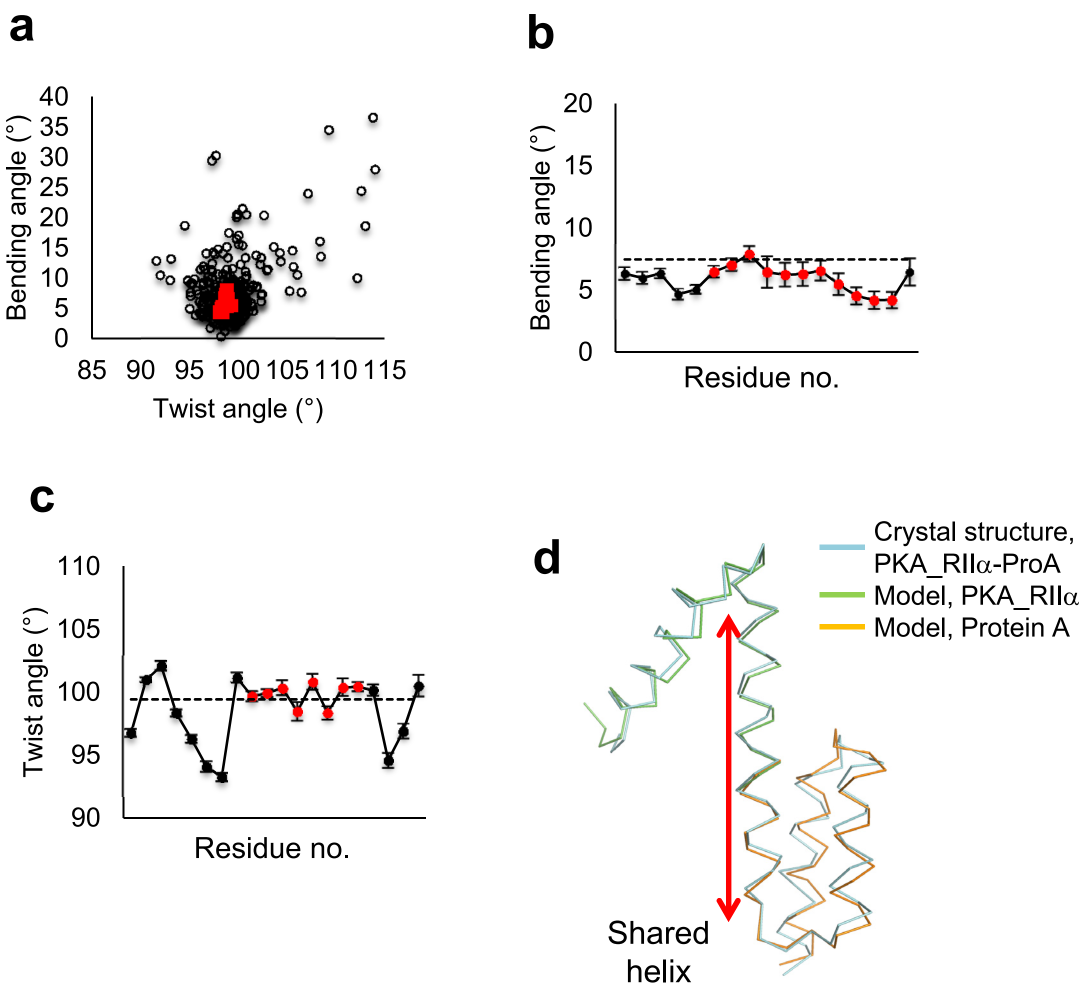

Supplementary Figure 5 | Structural analysis of the connecting helix in the PKA_RIIα-protein A fusion protein. (**a**) Plot of the twist and bending angles of 450 alpha helices in 45 standard protein structures [1]. The angles of the connecting helices in the asymmetric unit of the PKA_RIIα-protein A crystal are marked with red squares. The crystal contains twelve protein molecules in the asymmetric unit. (**b**) Bending angle distribution of the connecting helix. The shared part of the connecting helix is marked with red dots. The bending angle of the ideal alpha helix is drawn as a broken line. (**c**) Twist angle distribution of the connecting helix. The shared part of the connecting helix is marked with red dots. The twist angle of the ideal alpha helix is drawn as a broken line. (**d**) Comparison of the model and crystal structures of the PKA_RIIα-protein A fusion. The PKA_RIIα parts of the structures are aligned.

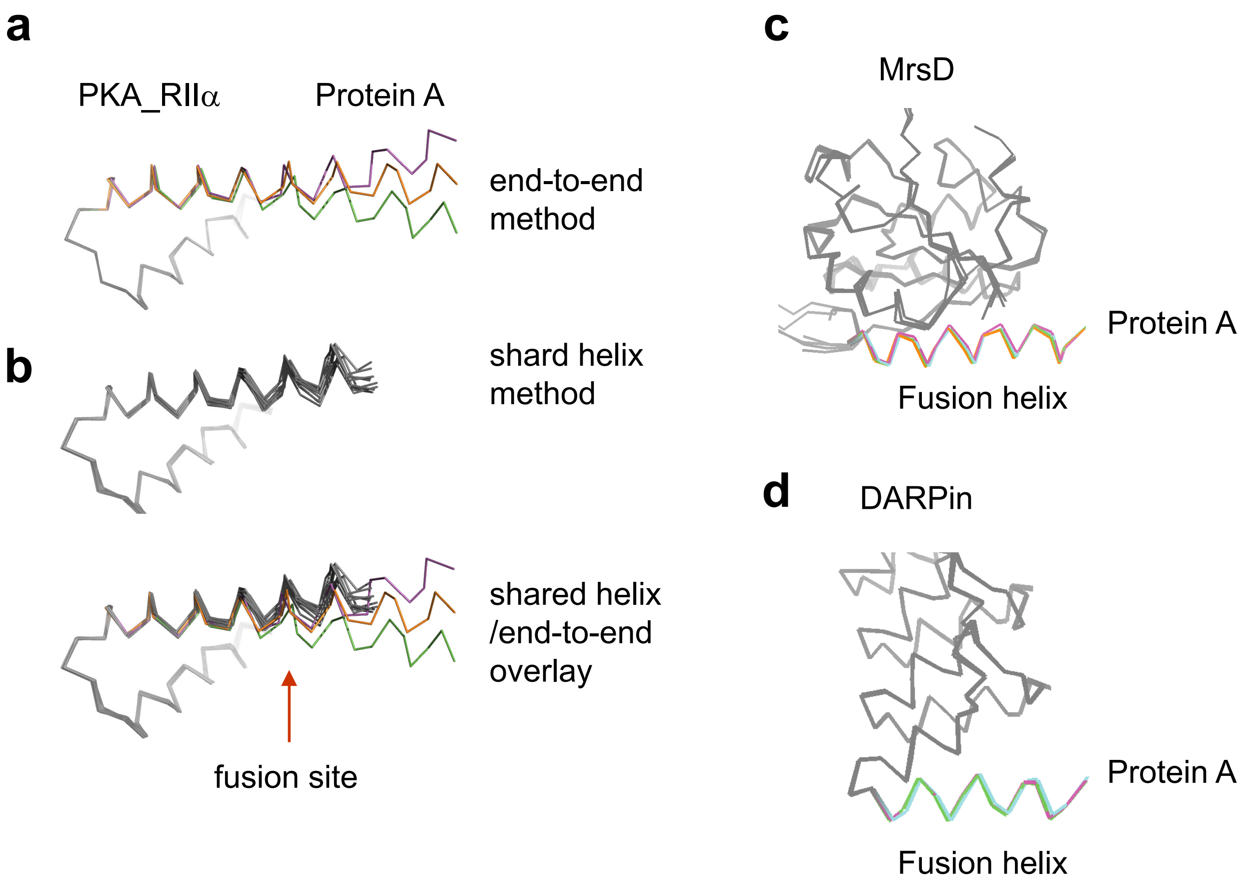

Supplementary Figure 6 | Structural flexibility of connecting helices in the PKA_RIIα-protein A (end-to-end) fusion protein. (a) Structural comparison of connecting helices of the three end-to-end fusion proteins in the crystal asymmetric unit. The crystal contains four fusion proteins in the asymmetric unit. The Protein A part of one of the fusion proteins does not have a clear electron density, presumably due to high structural flexibility at the fusion site (not shown in the figure). The PKA-RIIα regions of the fusion proteins are superimposed and the Cα traces are shown in grey. The protein A parts of the connecting helices are colored in magenta, orange and green, respectively. Structures of the protein A apart from the connecting helix are omitted for clarity. (b) Structural comparison of connecting helices of the twelve PKA_RIIα-protein A fusion proteins in the crystal asymmetric unit. The fusion proteins are connected by the shared helix method, as shown in Fig. 2b. The PKA-RIIα regions of the fusion proteins are superimposed and the Cα traces are shown in grey. The fusion proteins connected by the shared helix method and the end-to-end fusion methods are superimposed for comparison in the lower panel. Structures of protein A apart from the connecting helix are omitted for clarity. (c) Structural comparison of the connecting helices of the four MrsD-protein A fusion proteins in the crystal asymmetric unit that are ligated by the shared helix method (Fig. 1). The MrsD regions of the fusion proteins are superimposed and the Cα traces of the fusion proteins are shown in grey. The protein A parts of the connecting helices are colored in cyan, magenta, orange and green, respectively. Structures of protein A except for the connecting helix are omitted for clarity. (d) Structural comparison of the connecting helices of the three DARPin-protein A fusion proteins in the crystal asymmetric unit that are ligated by the shared helix method (Fig. 2a). The DARPin regions of the fusion proteins are superimposed and the Cα traces of the fusion proteins are shown in grey. The protein A parts of the connecting helices are colored in cyan, magenta and green, respectively. Structures of protein A except for the connecting helix are omitted for clarity.

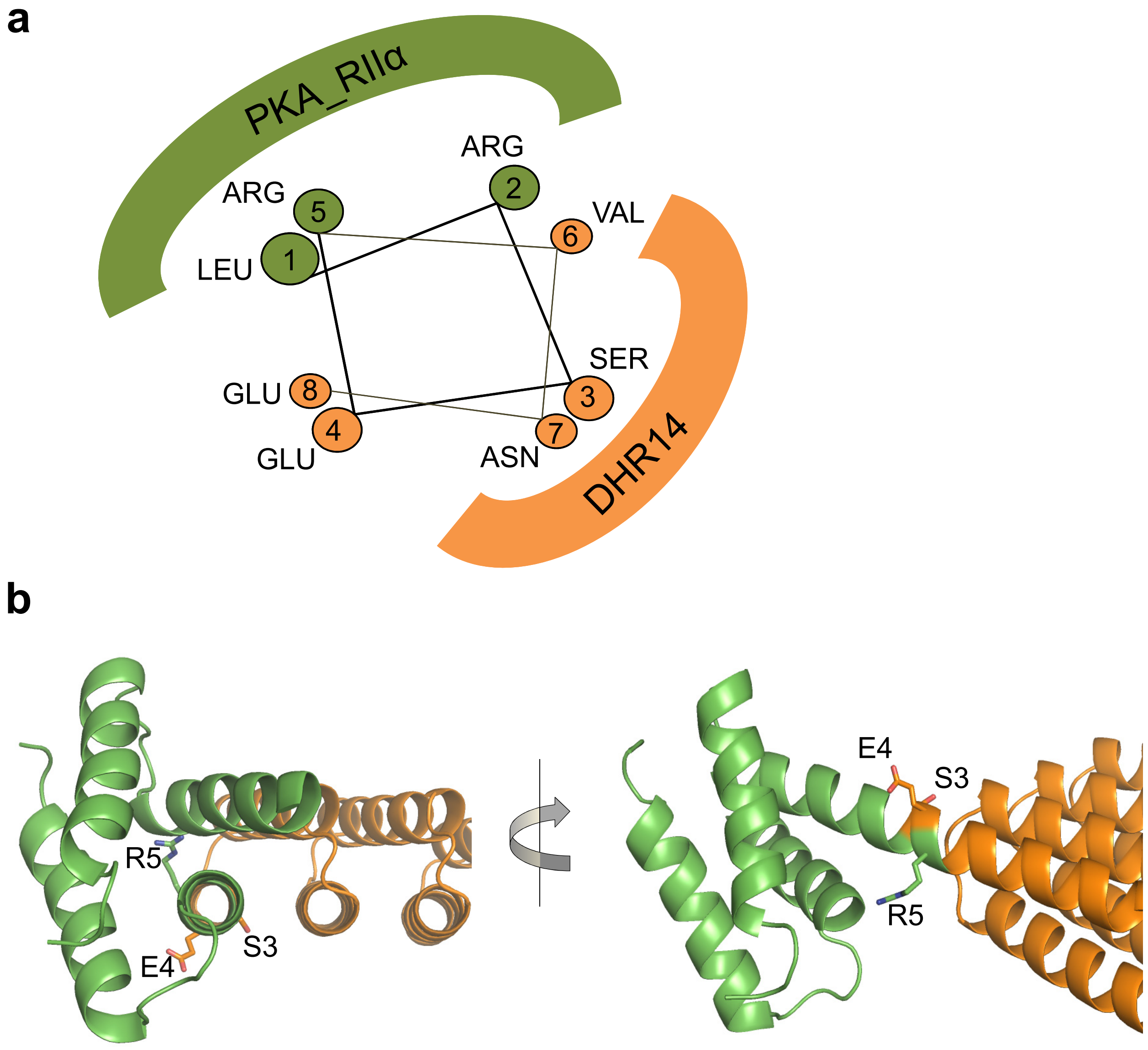

Supplementary Figure 7 | Structure of the connecting helix in the PKA_RIIα-DHR14 fusion. (**a**) Amino acid selection strategy. Amino acids of PKA_RIIα were selected for positions 1, 2 and 5 of the shared helix region, and those of DHR14 were selected for positions 3, 4, 6, 7 and 8 (Fig. 2c). (**b**) The crystal structure of the PKA_RIIα-DHR14 fusion protein. The structure of the connecting helix region is shown. The side chains of the shared helix region are drawn as sticks.

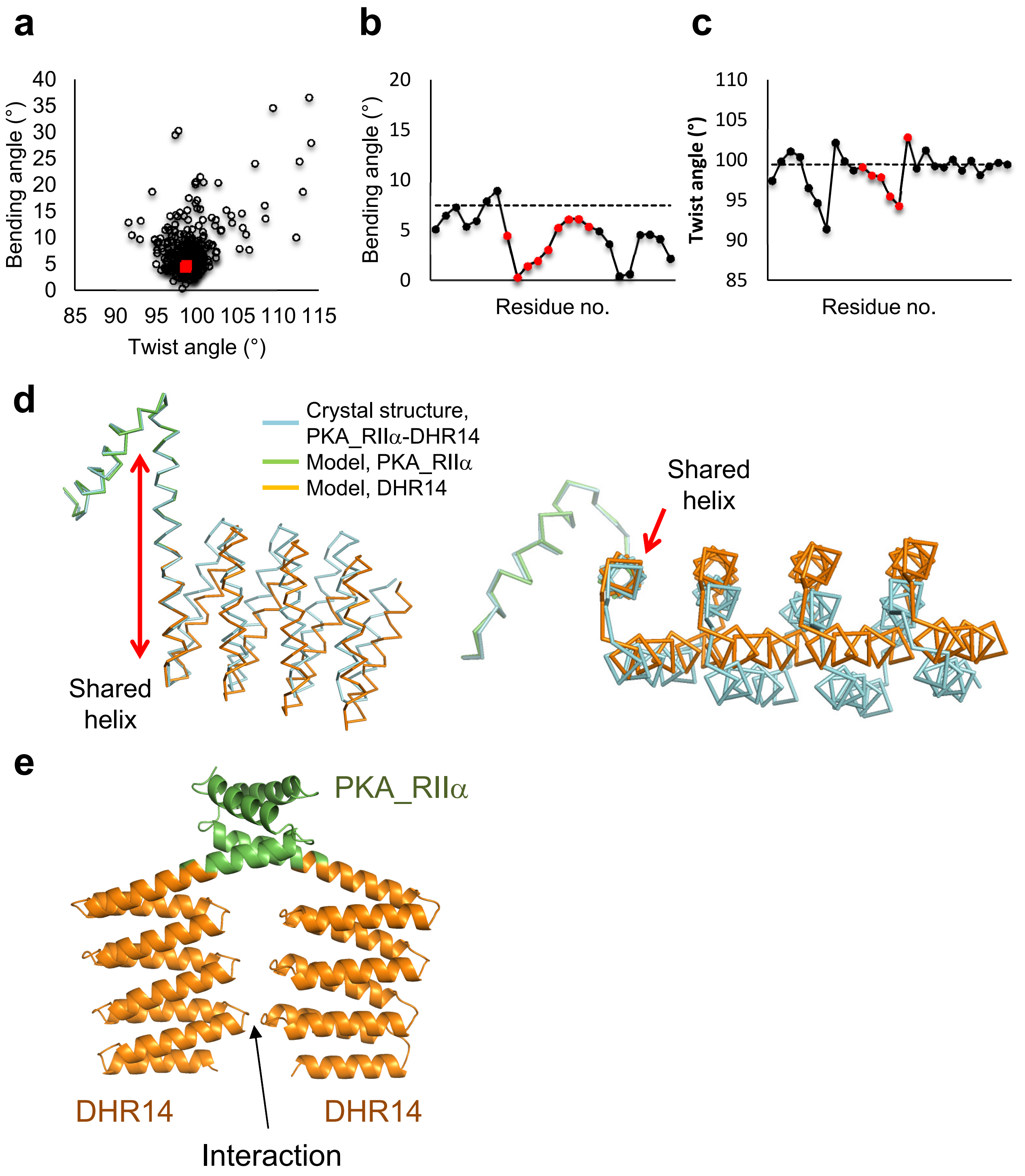

Supplementary Figure 8 | Structural analysis of the connecting helix in the PKA_RIIα-DHR14 fusion protein. (**a**) Plot of the twist and bending angles of 450 alpha helices in 45 standard protein structures [1]. The angles of the connecting helices in the asymmetric unit of the PKA_RIIα-DHR14 crystal are marked with red squares. The crystal contains two molecules in the asymmetric unit. (**b**) Bending angle distribution of the connecting helix. The shared part of the connecting helix is marked with red circles or squares. The bending angle of the ideal alpha helix is drawn as a broken line. (**c**) Twist angle distribution of the connecting helix. The shared part of the connecting helix is marked with red dots. The twist angle of the ideal alpha helix is drawn as a broken line. (**d**) Comparison of the model and crystal structures of the PKA_RIIα-DHR14 fusion. The PKA_RIIα parts of the structures are aligned. (e) The region of the opportunistic interaction between the two DHR14 domains is marked with an arrow.

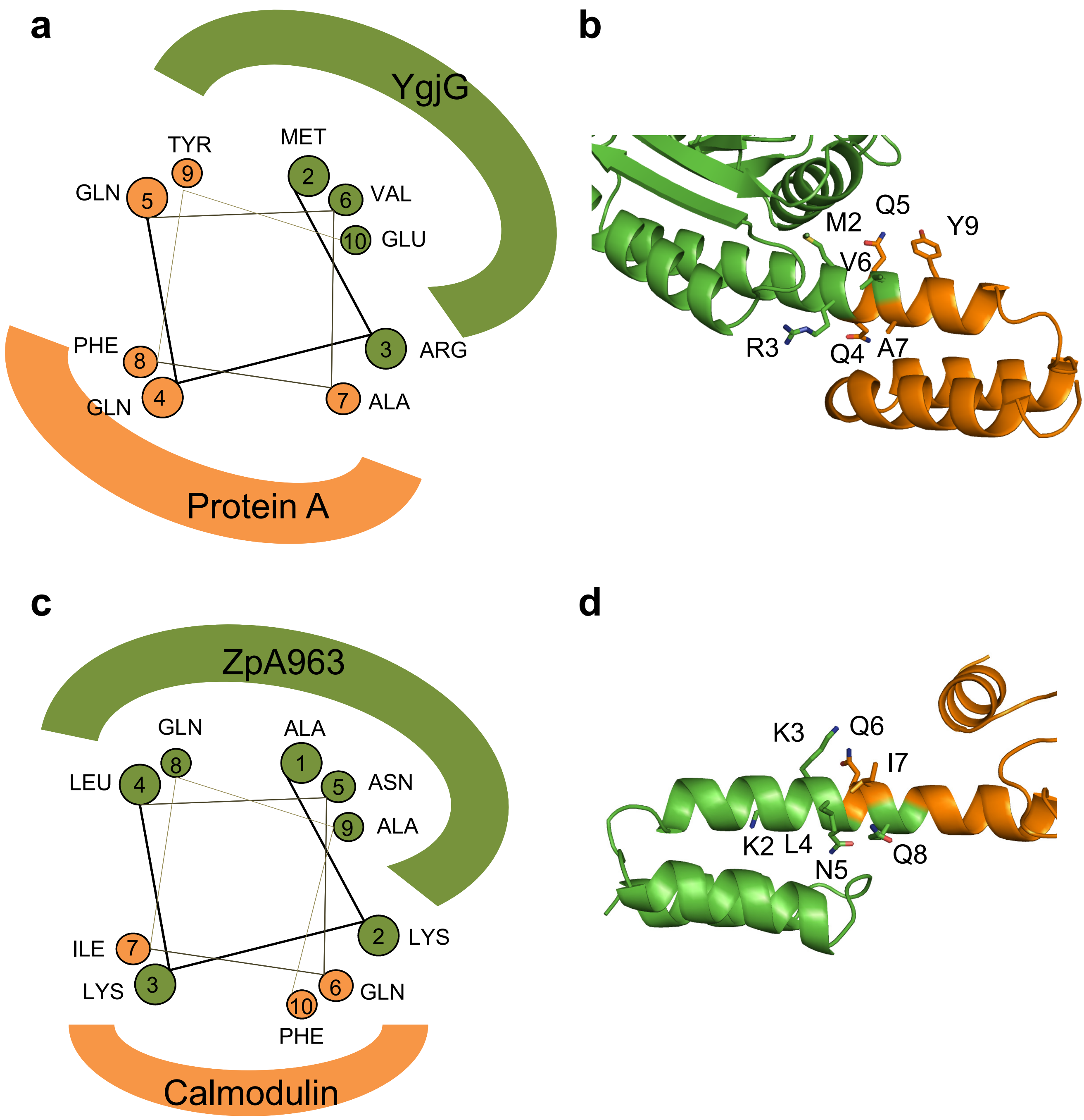

Supplementary Figure 9 | Structure of the connecting helix in the YgjG-protein A and ZpA963-calmodulin fusions. (**a**) Amino acid selection strategy. Amino acids of YgjG were selected for positions 2, 3, 6 and 10, and those of Protein A were selected for positions 4, 5, 7, 8 and 9 (Fig. 3a). (**b**) The crystal structure of the YgjG-protein A fusion protein. The structure of the connecting helix region is shown. (**c**) Amino acid selection strategy. Amino acids of ZpA963 were selected for positions 1, 2, 3, 4, 5, 8 and 9 (Fig. 3b). The lysine in position 3 points to the solvent area and is not important for the structural stability of the connecting helix. Amino acids of calmodulin were selected for positions 6, 7 and 10. (**d**) The crystal structure of the ZpA963-calmodulin fusion protein. The structure of the connecting helix area is shown. The side chains of the shared region are drawn as sticks.

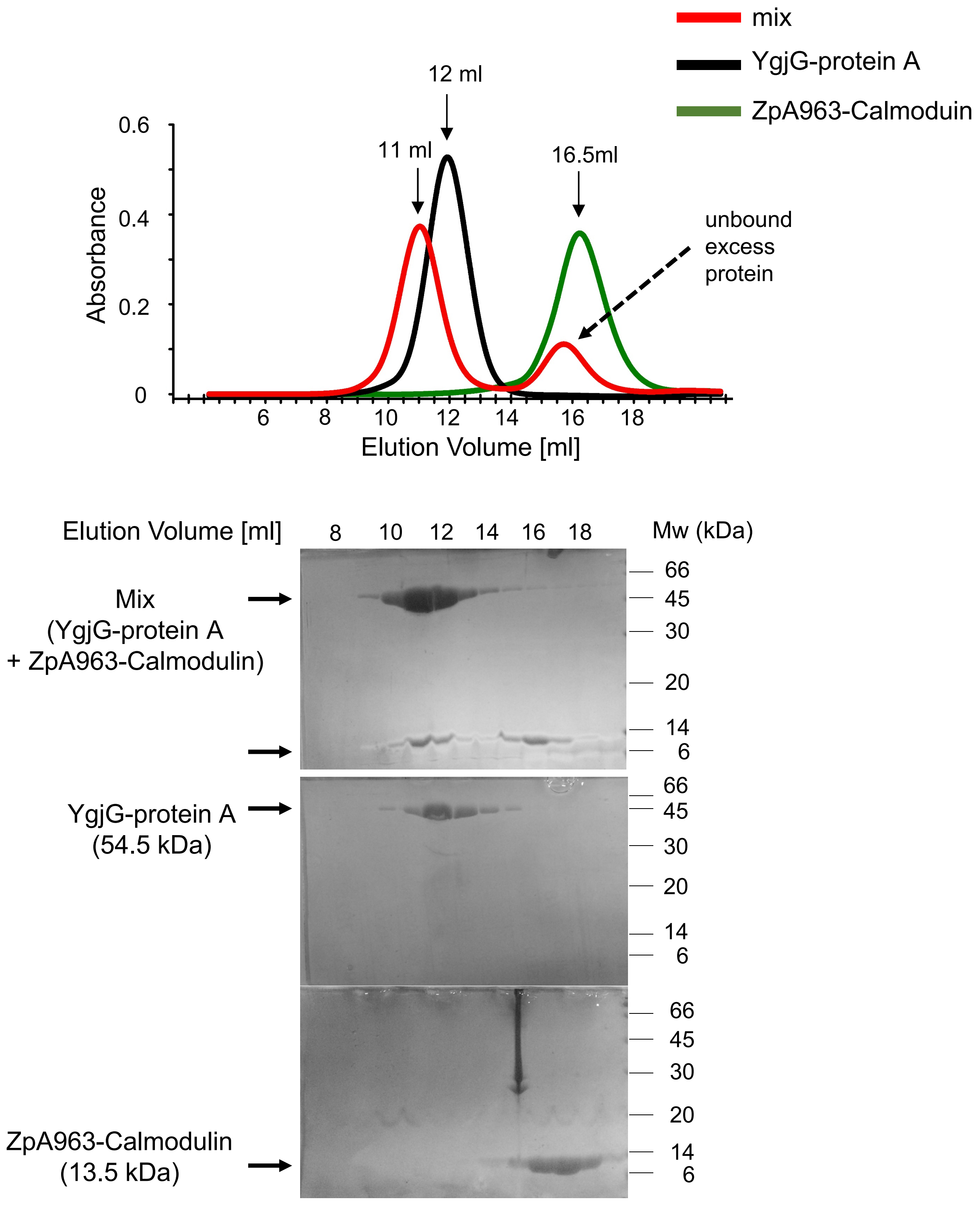

Supplementary Figure 10 | Binding of YgjG-protein A to the ZpA963-calmodulin fusion proteins. YgjG-protein A, the ZpA963-calmodulin fusion proteins and a mixture of these proteins were incubated in a buffer containing 20 mM Tris HCl pH 8.0, 200 mM NaCl and 5 mM $CaCl_2$ and analyzed by Superdex 200 gel-filtration chromatography. The ultraviolet (UV) absorption profiles measured at 280 nm are plotted. Twelve 1.0 ml fractions corresponding to elution volumes ranging from 8.0 ml to 19.0 ml were separated by SDS-PAGE and stained with Coomassie Brilliant Blue, and the results are shown in the lower panels. The two fusion proteins formed a stable complex and were eluted together in the gel-filtration chromatography results.

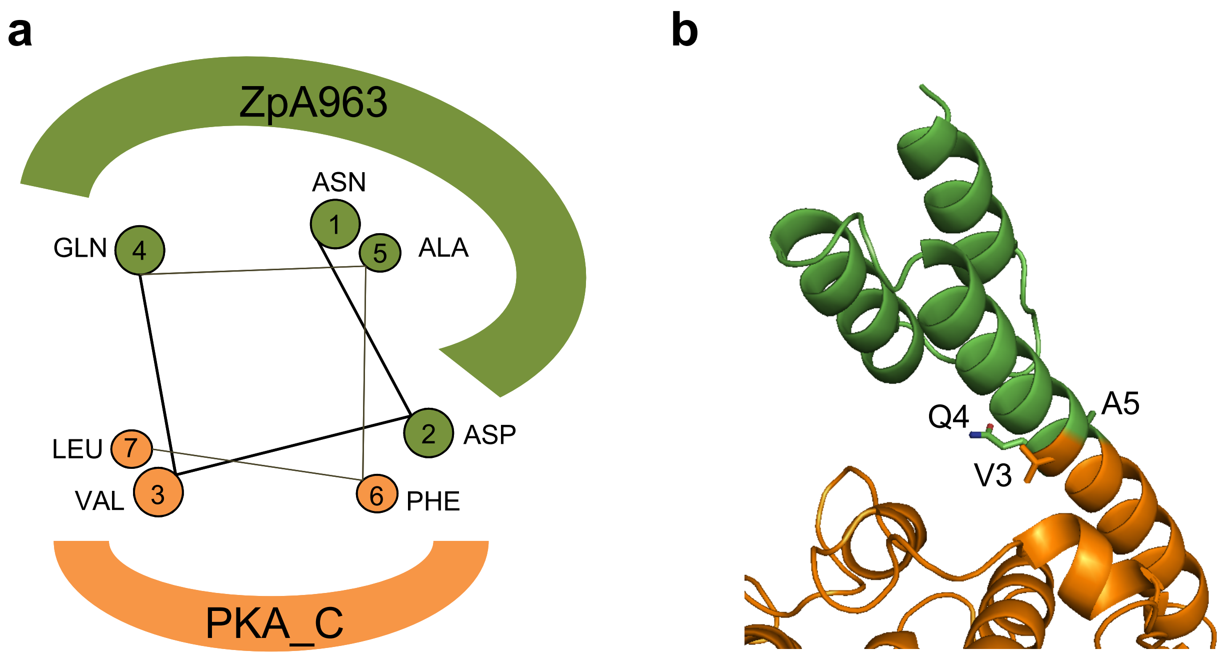

Supplementary Figure 11 | Structure of the connecting helix in the ZpA963-PKA_C fusion. (**a**) Amino acid selection strategy. Amino acids of ZpA963 were selected for positions 1, 2, 4 and 5. Amino acids of the catalytic domain of PKA (PKA_C) were selected for positions 3, 6 and 7 (Fig. 4a). (**b**) The crystal structure of the ZpA963-PKA_C fusion protein. The structure of the connecting helix region is shown. The side chains of the shared region are drawn as sticks.

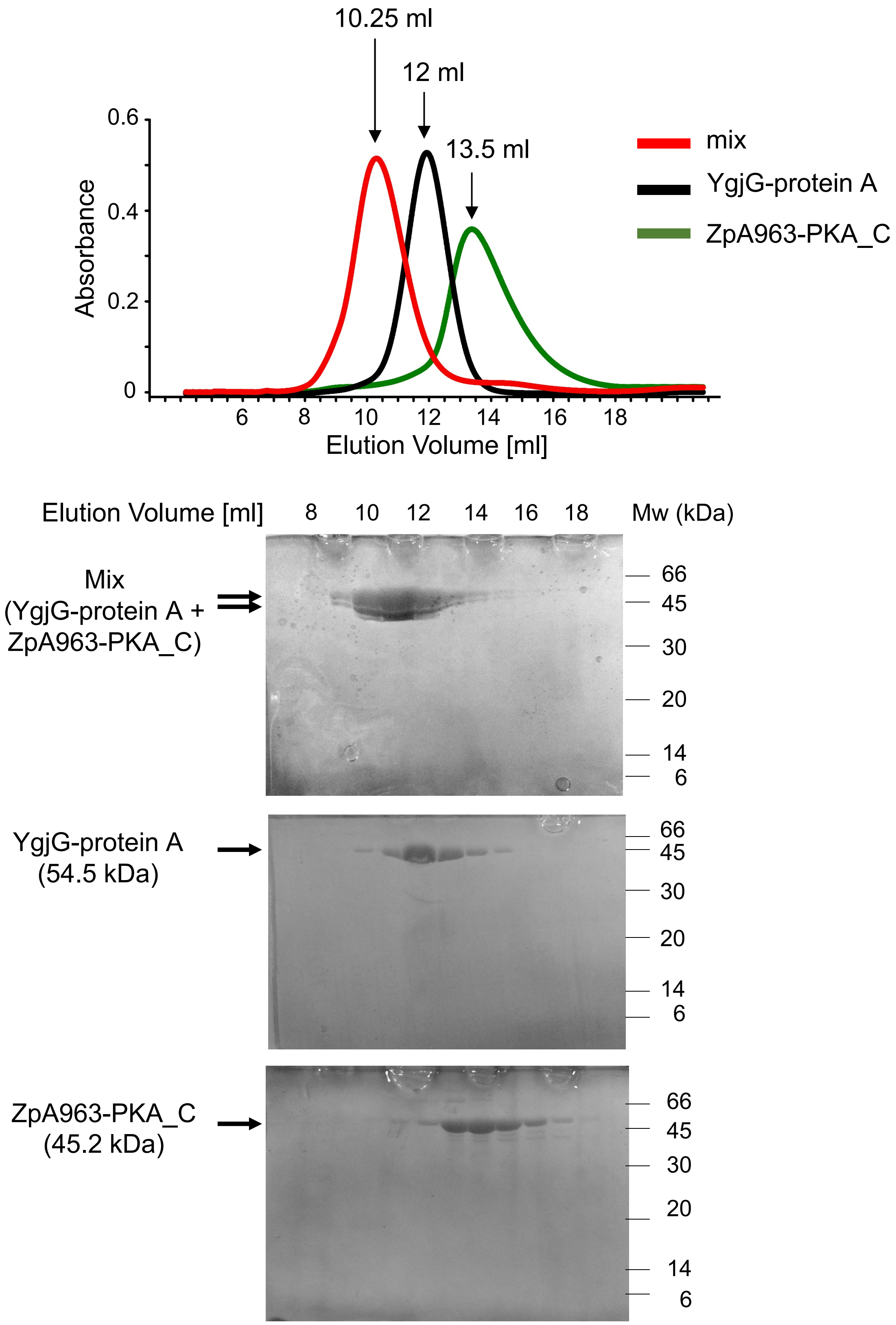

Supplementary Figure 12 | Binding of YgjG-protein A to ZpA963-PKA_C fusion proteins. YgjG-protein A, the ZpA963-PKC_C fusion proteins, and a mixture of these proteins were incubated in a buffer containing 20 mM Tris HCl pH 8.0 and 200 mM NaCl and analyzed by Superdex 200 gel-filtration chromatography. The UV absorption profiles measured at 280 nm are plotted. Twelve 1.0 ml fractions corresponding to elution volumes ranging from 8.0 ml to 19.0 ml were separated by SDS-PAGE and stained with Coomassie Brilliant Blue, and the results are shown in the lower panels. The two fused proteins formed a stable complex and were eluted together in the gel-filtration chromatography results.

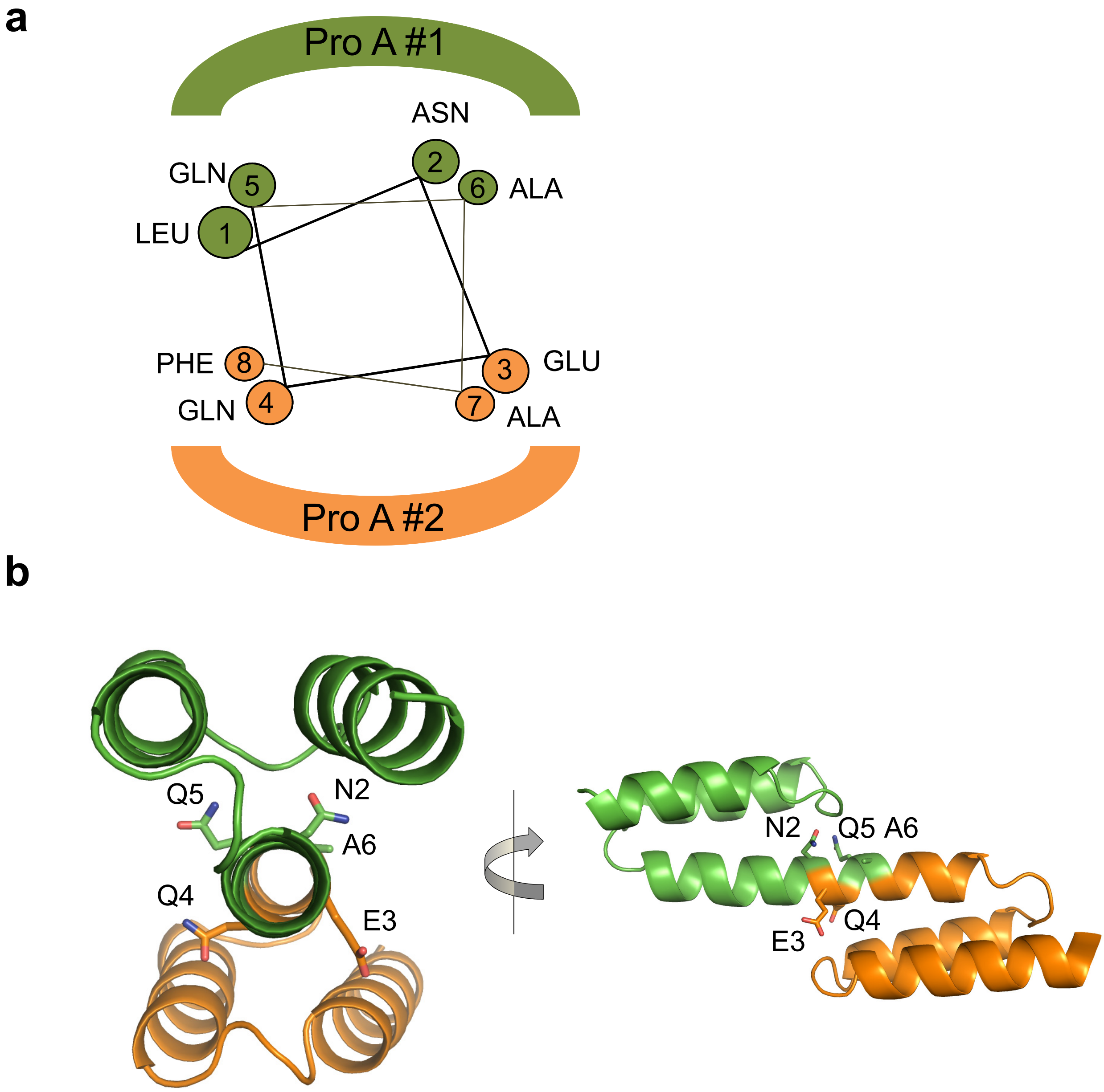

Supplementary Figure 13 | Structure of the connecting helix in the protein A repeat proteins. (**a**) Amino acid selection strategy. Amino acids of the first protein A were selected for positions 1, 2, 5 and 6, and those of the second protein A were selected for positions 3, 4, 7 and 8 (Fig. 5a). (**b**) The crystal structure of the protein A-protein A fusion in the repeat proteins. The structure of the connecting helix area is shown. The side chains of the shared region are drawn as sticks.

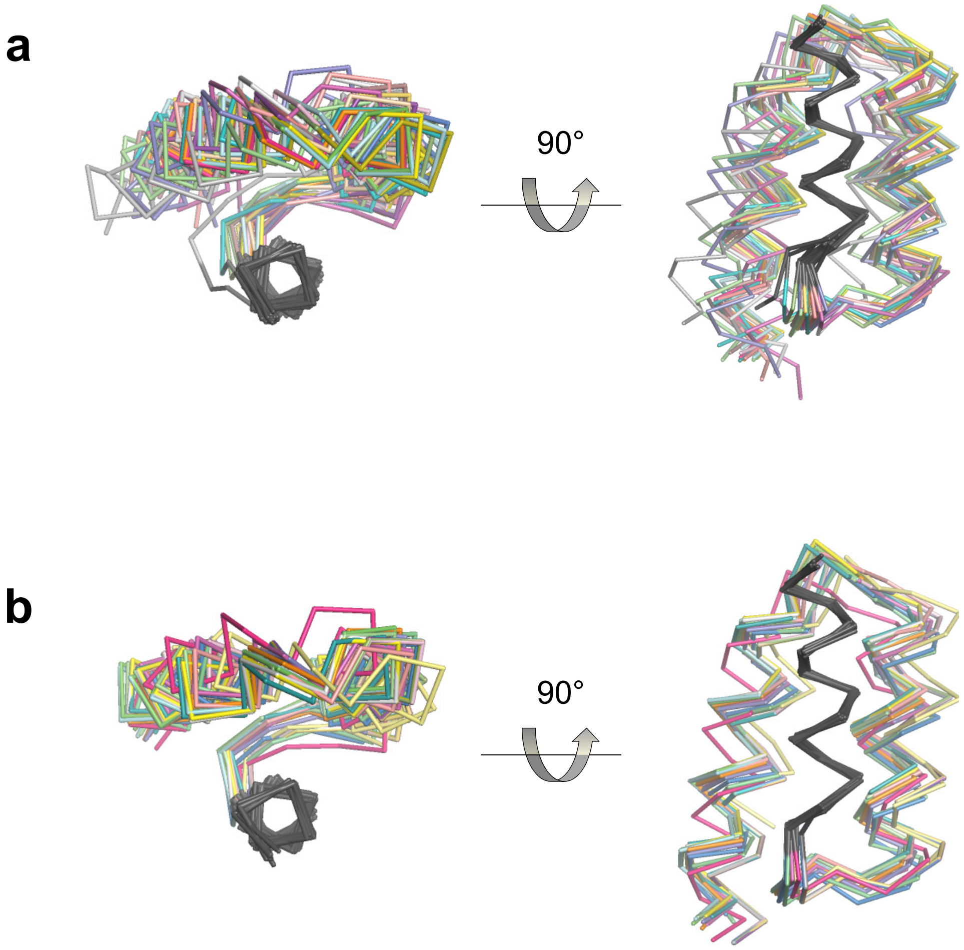

Supplementary Figure 14 | Structural flexibility of protein A. (**a**) Comparison of the protein A structures in the MrsD-Pro A, DARPin-Pro A, PKA_RIIα-Pro A, protein A 3-, 4- and 5-repeat proteins. N-terminal alpha helices of the protein A structures are aligned for comparison. (**b**) Comparison of the previously reported structures of the protein A domain. The structures were extracted from PDB coordinate files 4ZNC, 4WWI, 4ZMD, 1DEE, 5CBN, 5CBO, 5COC and 5EWX.

a

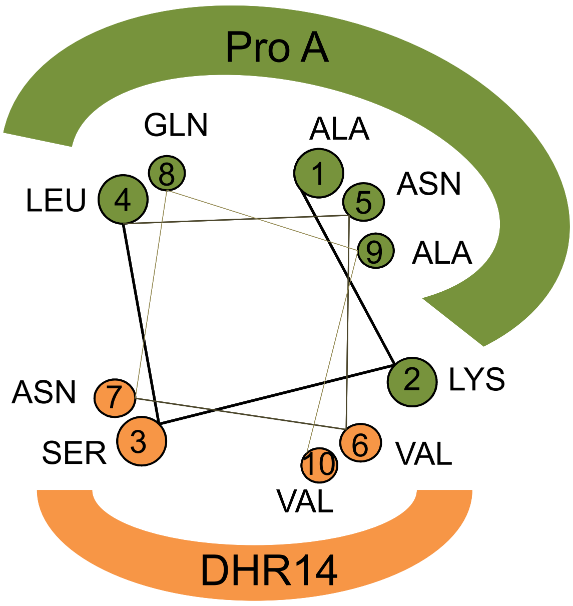

b

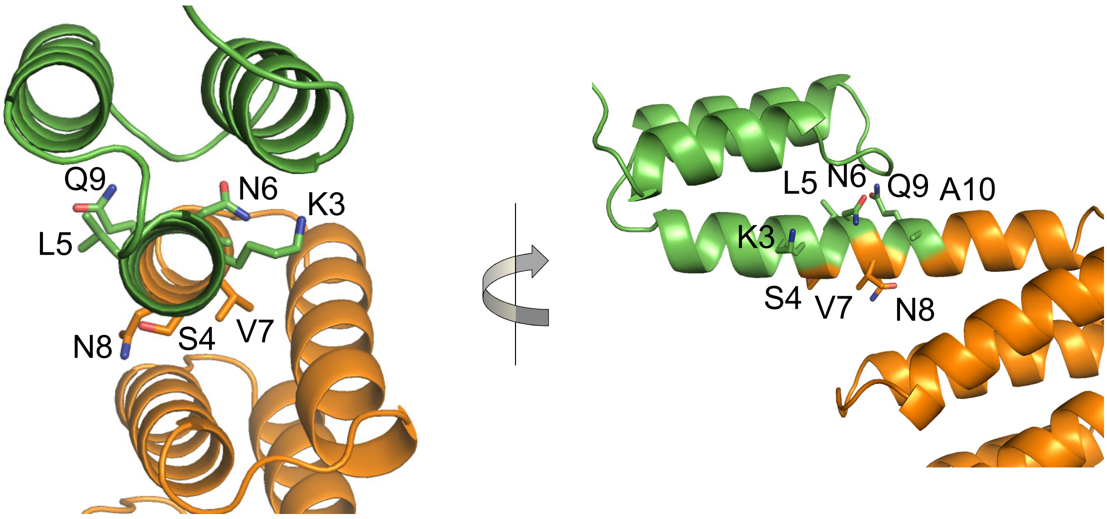

Supplementary Figure 15 | Structure of the connecting helix in protein A-DHR14 in the mixed repeat protein. (**a**) Amino acid selection strategy. Amino acids of protein A were selected for positions 1, 2, 4, 5, 8 and 9, and those of DHR14 were selected for positions 3, 6, 7 and 10 (Fig. 6a). (**b**) The crystal structure of the protein A-DHR14 fusion in the mixed repeat protein. The structure of the connecting helix area is shown. The side chains of the shared region are drawn as sticks.

a

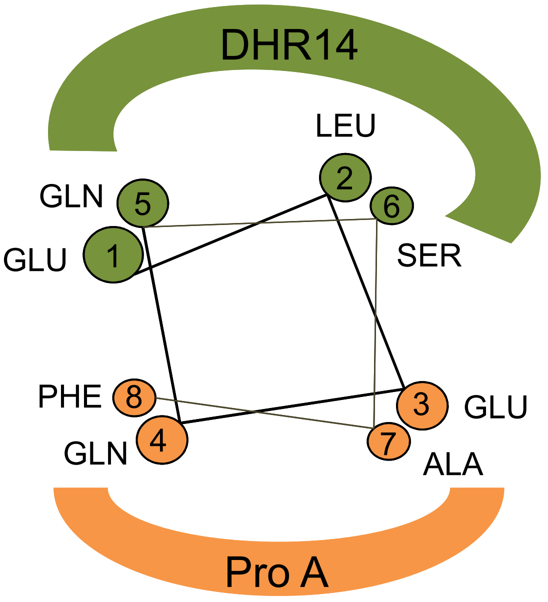

b

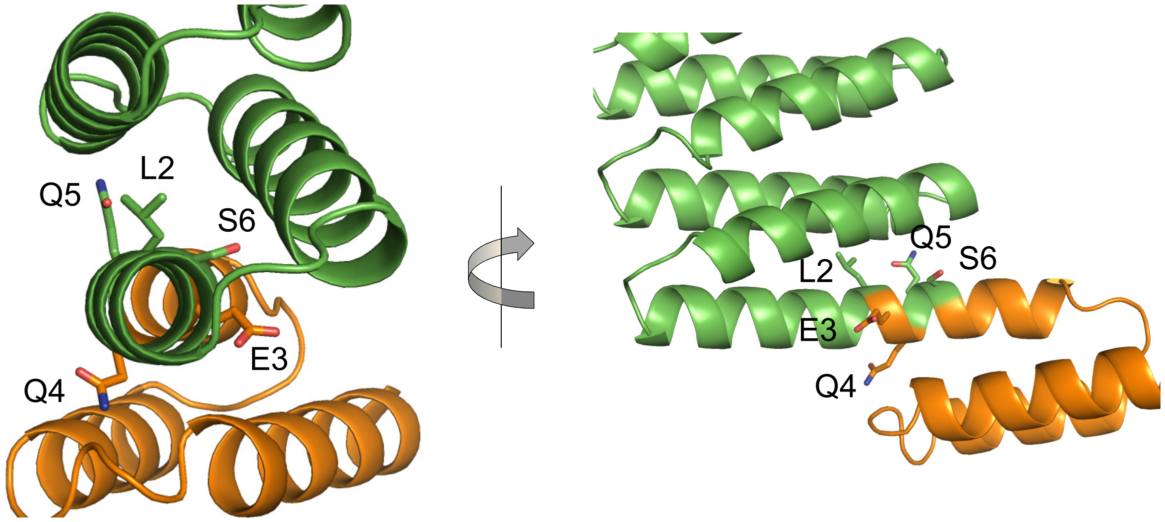

Supplementary Figure 16 | Structure of the connecting helix in the DHR14-protein A in the mixed repeat protein. (**a**) Amino acid selection strategy. Amino acids of DHR14 were selected for positions 1, 2, 5 and 6, and those of protein A were selected for positions 3, 4, 7 and 8 (Fig. 6b). (**b**) The crystal structure of the DHR14-protein A fusion in the mixed repeat protein. The structure of the connecting helix region is shown. The side chains of the shared region are drawn as sticks.

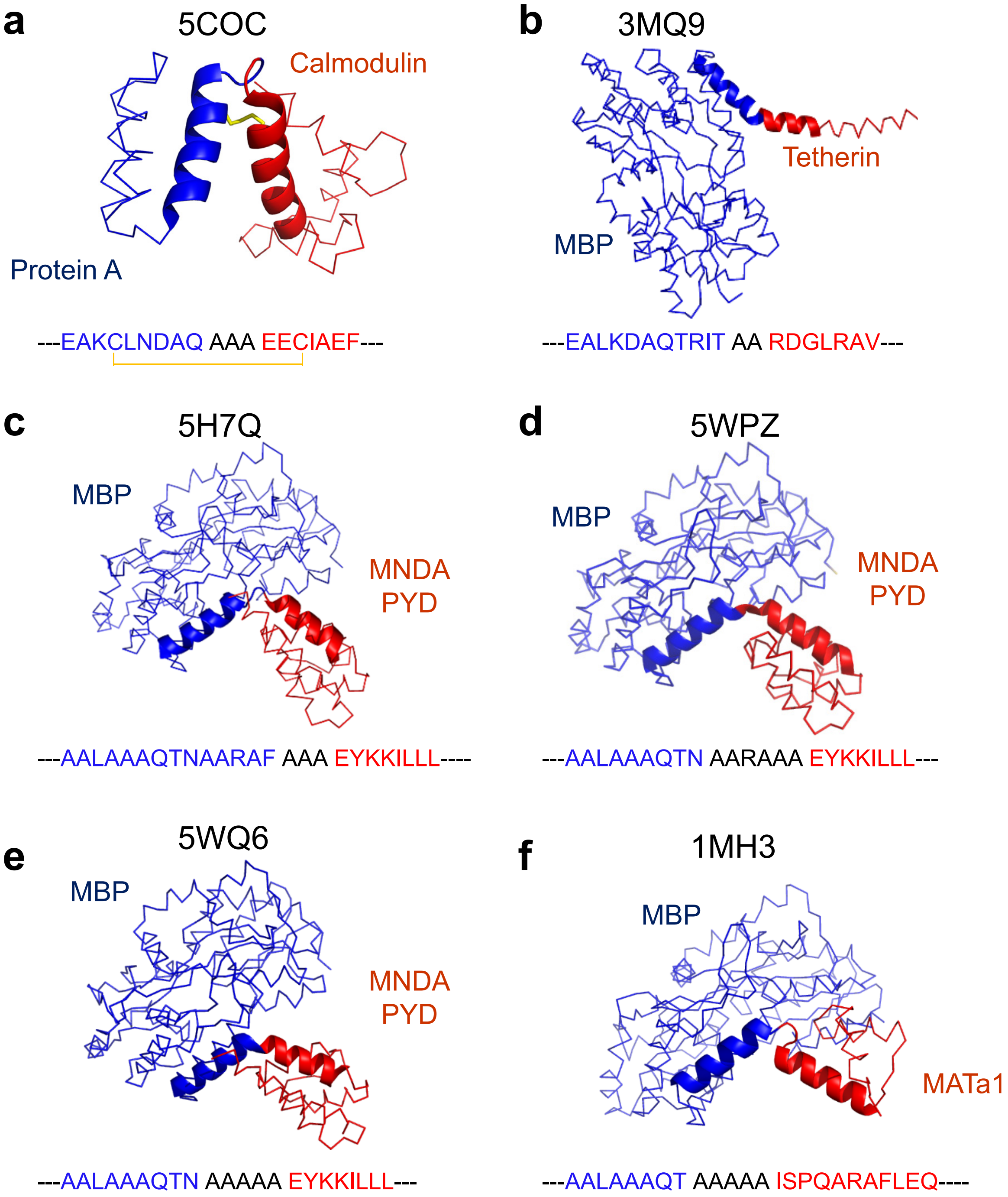

Supplementary Figure 17 | Crystal structures of fusion proteins joined by the end-to-end ligation of alpha helices. Structures of the fused proteins are shown in blue and red, respectively. PDB codes are written above the crystal structures. The fusion alpha helices are highlighted with ribbons. Amino acid sequences of the first proteins, the alanine linkers and the second proteins at the fusion sites are written underneath in blue, black and red, respectively. A disulfide bridge connecting the fusion helices of the 5COC structure is shown by the yellow line.

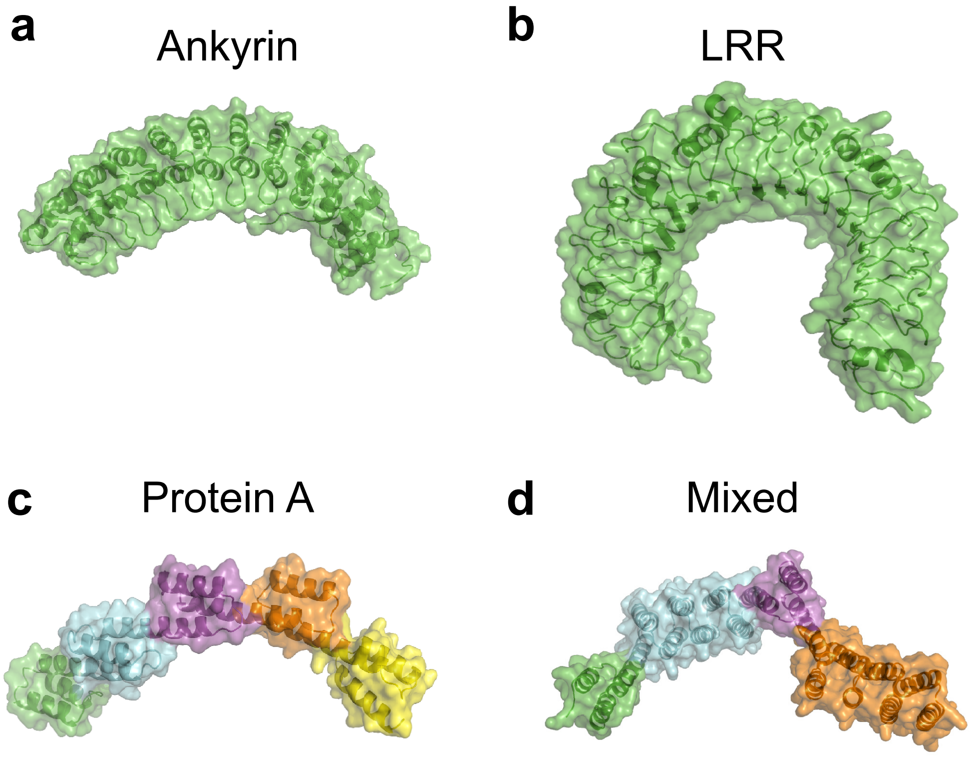

Supplementary Figure 18 | Curvature of repeat family proteins. (**a**) Crystal structure of a human ankyrin 1 (PDB code, 1N11). (**b**) Crystal structure of the LRR family protein, TLR2 (PDB code, 3A79). (c) The crystal structure of the protein A repeat protein. (d) The crystal structure of the protein A-DHR14 mixed repeat protein.

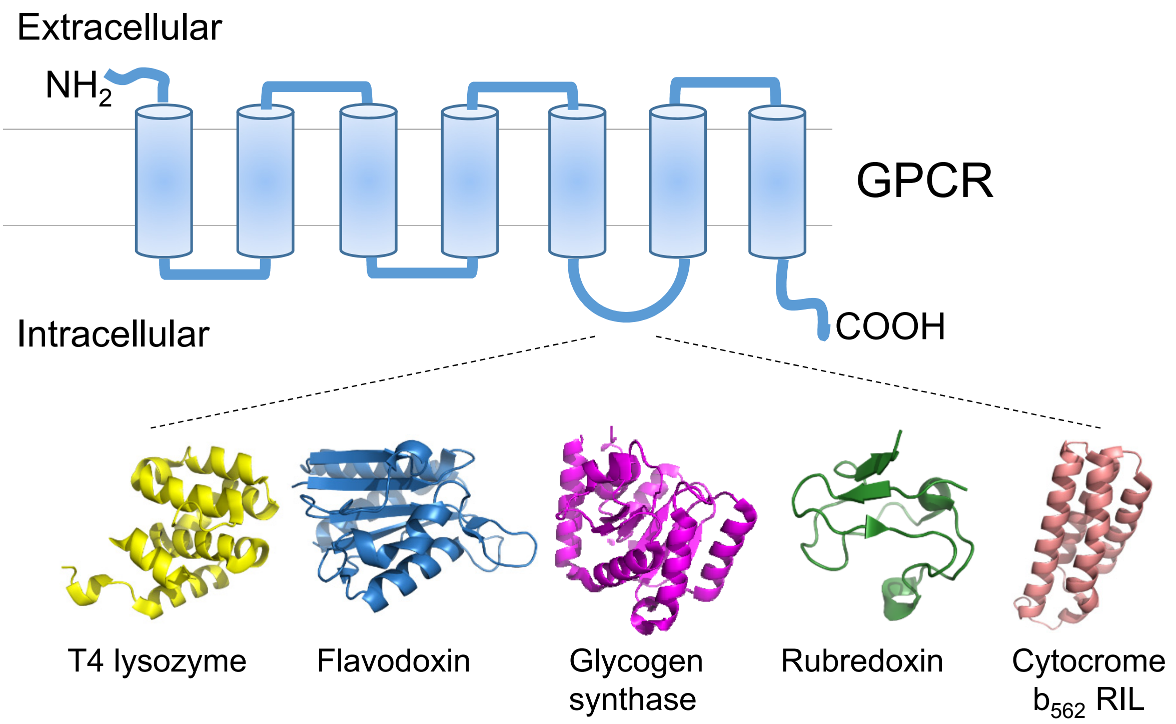

4AMI	4U15	3ZPQ	5TGZ	4ZJ8	4MBS	5IUA	4N6H
4DJH	3QAK	5CGC		4S0V	4XNV	5K2A	4QIM
3D4S	5D5B	4DAJ		4ZJC	4XNW	5K2B	4YAY
5CXV	3NY9	4EJ4		5U09		4IAQ	5K2D
3OE6	4PHU	4GBR				5L7D	4Z34
5GLI	4GRV	3UON				4PY0	5L7I
4LDO	2VT4	5DSG				4IAR	4Z36
3SN6	3PBL	4U16				4Z35	5IU4
5D5A	3NYA	3PDS				4BVN	4L6R
4RWS	3ZPR	5CSD				5IU7	5IUA
3OE0	5F8U	3EML				4EIY	5K2C
3NY8	3ODU	4OO9				4EA3	4IB4
4Z9G	3ZPQ	5D6L				4QIN	4NTJ
3VW7	3ODU	5EE7				5IU4	4PXZ
4U14	4K5Y	2RH1				4O9R	4N4W
3V2Y	4AMJ	3OE9				5IUB	4NC3
3OE8	3V2W	4L0|					
4XES	4DKL	3RZE					
4ZWJ	5GLH						

Supplementary Figure 19 | Examples of proteins inserted into a flexible loop of a GPCR. Figure illustrating the insertion of five proteins into a flexible internal loop of a prototypical GPCR represented as a schematic plot. All proteins other than Rubredoxin have terminal alpha helices. PDB codes of the crystal structures of the GPCRs containing the inserted proteins are written underneath.

Supplementary Table 1 | Amino acid sequences of the fusion proteins ligated by the shared helix method.

Fusion proteins	Amino acid sequences
MrsD-proA	GSGGGGSMSISILKDKKLLIGICGSISSVGISSYLLYFKSFFKEIRVVMTKTAEDLIPAHTVS<br>YFCDHVYSEHGENGKRHSHVEIGRWADIYCIIPATANILGQTANGVAMNLVATTVLAHPH<br>NTIFFPNMNDLMWNKTVVSRNIEQLRKDGHIVIEPVEIMAFEIATGTRKPNRGLITPDKALL<br>**AIEQGF**AFYEILHLPNLNEEQRNAFIQSLKDDPSQSANLLAEAKKLNDAQAPK
DARPin-proA	GSHMDLGRKLLEAARAGQDDEVRILMANGADVNAADNTGTTPLHLAAYSGHLEIVEVLL<br>KHGADVDASDVFGYTPLHLAAYWGHLEIVEVLLKNGADVNAMDSDGMTPLHLAAKWGY<br>LEIVEVLLKHGAVNAQDKFGKTAFDISIDNGNE**DLAQIL**AFYEILHLPNLNEEQRNAFIQSL<br>KDDPSQSANLLAEAKKLNDAQA
PKA_RIIα-proA	GSPWQIPPGLTELLQGYTVEVLRQQPPDLVEFAVEYFT**RLRQQR**AFYEILHLPNLNEEQR<br>NAFIQSLKDDPSQSANLLAEAKKLNDAQAPK
PKA_RIIα-DHR14	GSHMQIPPGLTELLQGYTVEVLRQQPPDLVEFAVEYFTRL**RSER**VNERVKQLAEKAKEA<br>TDKEEVIEIVKELAELAKQSTDSELVNEIVKQLAEVAKEATDKELVIYIVKILAELAKQSTDS<br>ELVNEIVKQLAEVAKEATDKELVIYIVKILAELAKQSTDSELVNEIVKQLEEVAKEATDKELV<br>EHIEKILEELKKQSTD
YgjG-proA	GSHMSASALACSAHALNLIEKRTLDHEEMKALNREVIEYFKEHVNPGFLEYRKSVTAGGD<br>YGAVEWQAGSLNTLVDTQGQEFIDCLGGFGIFNVGHRNPVVVSAVQNQLAKQPLHSQE<br>LLDPLRAMLAKTLAALTPGKLKYSFFCNSGTESVEAALKLAKAYQSPRGKFTFIATSGAFH<br>GKSLGALSATAKSTFRKPFMPLLPGFRHVPFGNIEAMRTALNECKKTGDDVAAVILEPIQ<br>GEGGVILPPPGYLTAVRKLCDEFGALMILDEVQTGMGRTGKMFACEHENVQPDILCLAK<br>ALGGGVMPIGATIATEEVFSVLFDNPFLHTTTFGGNPLACAAALATINVLLEQNLPAQAEQ<br>KGDMLLDGFRQLAREYPDLVQEARGKGMLMAIEFVDNEIGYNFASEMFRQRVLVAGTLN<br>NAKTIRIEPPLTLTIEQCELVIKAARKALA**AMRQQVAFYE**ILHLPNLNEEQRNAFIQSLKDD<br>PSQSANLLAEAKKLNDAQAPK
ZpA963-calmodulin	GSHMKFNKETQEASWEIFTLPNLNGRQVAAFISSLLDDPSQSANLLAEA**KKLNQIQA**FKE<br>AFSLFDKDGDGTITTKELGTVMRSLGQNPTEAELQDMINEVDADGNGTIDFPEFLTMMA<br>RKM
ZpA963-PKA_C	GSHMVDNKFNKETQEASWEIFTLPNLNGRQVAAFISSLLDDPSQSANLLAEAKKLND**VQ**<br>**A**FLAKAKEDFLKKWETPSQNTAQLDQFDRIKTLGTGSFGRVMLVKHKESGNHYAMKILD<br>KQKVVKLKQIEHTLNEKRILQAVNFPFLVKLEFSFKDNNLYMVMEYVAGGEMFSHLRRIG<br>RFSEPHARFYAAQIVLTFEYLHSLDLIYRDLKPENLLIDQQGYIQVTDFGFAKRVKGRTWT<br>LCGTPEYLAPEIILSKGYNKAVDWWALGVLIYEMAAGYPPFFADQPIQIYEKIVSGKVRFPS<br>HFSSDLKDLLRNLLQVDLTKRFGNLKNGVNDIKNHKWFATTDWIAIYQRKVEAPFIPKFKG<br>PGDTSNFDDYEEEEIRVSINEKCGKEFTEF
proA 3 repeats	GSHMKFNKEQQNAFYEILHLPNLNEEQRNAFIQSLKDDPSQSANLLAEAKK**LNEQQA**AF<br>YEIL**S**LPNLNEEQRNAFIQSLKDDPSQSANLLAEAKK**LNEQQA**AFYEILHLPNLNEEQRNA<br>FIQSLKDDPSQSANLLAEAKKLNDAQAPK
proA 4 repeats	GSHMKFNKEQQNAFYEILHLPNLNEEQRNAFIQSLKDDPSQSANLLAEAKK**LNEQQA**AF<br>YEIL**S**LPNLNEEQRNAFIQSLKDDPSQSANLLAEAKK**LNEQQA**AFYEILHLPNLNEEQRNA<br>FIQSLKDDPSQSANLLAEAKK**LNEQQA**AFYEILHLPNLNEEQRNAFIQSLKDDPSQSANLL<br>AEAKKLNDAQAPK
proA 5 repeats	GSHMKFNKEQQNAFYEILHLPNLNEEQRNAFIQSLKDDPSQSANLLAEAKK**LNEQQA**AF<br>YEIL**S**LPNLNEEQRNAFIQSLKDDPSQSANLLAEAKK**LNEQQA**AFYEILHLPNLNEEQRNA<br>FIQSLKDDPSQSANLLAEAKK**LNEQQA**AFYEILHLPNLNEEQRNAFIQSLKDDPSQSANLL<br>AEAKK**LNEQQA**AFYEILHLPNLNEEQRNAFIQSLKDDPSQSANLLAEAKKLNDAQAPK
proA-DHR14 2 repeats	GSHMKFNKEQQNAFYEILHLPNLNEEQRNAFIQSLKDDPSQSANLLAEA**KSLNVNQA**VK<br>QLAEKAKEATDKEEVIEIVKELAELAKQSTDSELVNEIVKQLAEVAKEATDKELVIYIVKILA<br>ELAKQSTDSELVNEIVKQLAEVAKEATDKELVIYIVKILAELAKQSTDSELVNEIVKQLEEVA<br>KEATDKELVEHIEKILE**ELEQQS**AFYEIL**S**LPNLNEEQRNAFIQSLKDDPSQSANLLAEA**KS**<br>**LNVNQA**VKQLAEKAKEATDKEEVIEIVKELAELAKQSTDSELVNEIVKQLAEVAKEATDKE<br>LVIYIVKILAELAKQSTDSELVNEIVKQLAEVAKEATDKELVIYIVKILAELAKQSTDSELVNEI<br>VKQLEEVAKEATDKELVEHIEKILEELKKQSTD

Amino acid sequences of the shared helix are written in red. Amino acids of protein A that were mutated for cloning are written in bold and underlined.

Supplementary Table 2 | Amino acid sequences of the end-to-end fusion proteins.

Fusion proteins	Amino acid sequences
PKA_RIIα-proA (end-to-end)	GSPWQIPPGLTELLQGYTVEVLRQQPPDLVEFAVEYFTRLREAR**NKEQQNAFYEILHLPNL NEEQRNAFIQSLKDDPSQSANLLAEAKKLNDAQAPK**
PKA_RIIα-DHR14 (end-to-end)	GSHMQIPPGLTELLQGYTVEVLRQQPPDLVEFAVEYFTRLREAR **DSEEVNERVKQLAEKAKEATDKEEVIEIVKELAELAKQSTDSELVNEIVKQLAEVAKEATD KELVIYIVKILAELAKQSTDSELVNEIVKQLAEVAKEATDKELVIYIVKILAELAKQSTDSELV EIVKQLEEVAKEATDKELVEHIEKILEELKKQSTD**
YgjG-proA (end-to-end)	GSHMSASALACSAHALNLIEKRTLDHEEMKALNREVIEYFKEHVNPGFLEYRKSVTAGGDY GAVEWQAGSLNTLVDTQGQEFIDCLGGFGIFNVGHRNPVVVSAVQNQLAKQPLHSQELLD PLRAMLAKTLAALTPGKLKYSFFCNSGTESVEAALKLAKAYQSPRGKFTFIATSGAFHGKSL GALSATAKSTFRKPFMPLLPGFRHVPFGNIEAMRTALNECKKTGDDVAAVILEPIQGEGGVIL PPPGYLTAVRKLCDEFGALMILDEVQTGMGRTGKMFACEHENVQPDILCLAKALGGGVMPI GATIATEEVFSVLFDNPFLHTTTFGGNPLACAAALATINVLLEQNLPAQAEQKGDMLLDGFR QLAREYPDLVQEARGKGMLMAIEFVDNEIGYNFASEMFRQRVLVAGTLNNAKTIRIEPPLTL TIEQCELVIKAARKALAAMRVSVEEAL**NKEQQNAFYEILHLPNLNEEQRNAFIQSLKDDPSQ SANLLAEAKKLNDAQAPK**

Amino acid sequences of the second proteins are written in bold.

Supplementary Table 3 | Summary of protein expression conditions.

fusion proteins	expression plasmid/ antibiotics	purification tag/affinity column
MrsD-proA	pET28a/kanamycin	MBP/amylose agarose
DARPin-proA	pET28a/kanamycin	6xHis/Ni-NTA agarose
PKA_RIIα-proA	pET3a/ampicillin	6xHis/Ni-NTA agarose
PKA_RIIα-proA (end-to-end)	pET3a/ampicillin	6xHis/Ni-NTA agarose
PKA_RIIα-DHR14	pET28a/kanamycin	6xHis/Ni-NTA agarose
PKA_RIIα-DHR14 (end-to-end)	pET28a/kanamycin	6xHis/Ni-NTA agarose
YgjG-proA	pETDuet-1/ampicillin	6xHis/Ni-NTA agarose
YgjG-proA (end-to-end)	pETDuet-1/ampicillin	6xHis/Ni-NTA agarose
ZpA963-calmodulin	pETDuet-1/ampicillin	6xHis/Ni-NTA agarose
ZpA963-PKA_C	pET28a/kanamycin	6xHis/Ni-NTA agarose
ProA 3 repeats	pET28a/kanamycin	6xHis/Ni-NTA agarose
ProA 4 repeats	pET28a/kanamycin	6xHis/Ni-NTA agarose
ProA 5 repeats	pET28a/kanamycin	6xHis/Ni-NTA agarose
ProA-DHR14 2 repeats	pET28a/kanamycin	6xHis/Ni-NTA agarose

Supplementary Table 4 | Summary of crystallization and crystal freezing conditions.

fusion proteins	crystallization solution	temp./ protein conc.	cryoprotectant*
MrsD-proA	0.94 M Na citrate pH 5.5	4°C / 24 mg/ml	30% glycerol
DARPin-proA	0.63 M NaK phosphate pH 9.5,	23°C / 5.4 mg/ml	30% ethylene glycol
PKA_RIIα-proA	0.68 M Na citrate pH 4.5, 0.1 M Na acetate pH 4.5	23°C / 5 mg/ml	30% glycerol
PKA_RIIα-proA (end-to-end)	2.16 M Na Formate 0.1 M Tris pH 8.5	23°C / 6.5 mg/ml	30% glycerol
PKA_RIIα-DHR14	12% PEG 4000 0.2M K chloride 0.01M Mg chloride 0.05M Na cacodylate pH 6.5	23°C / 10 mg/ml	30% glycerol
YgjG-proA/ ZpA963-calmodulin	20.7% PEG 300 99mM Ca chloride 0.1M HEPES pH 7.5	23°C / 22 mg/ml	9.3% PEG 300
YgjG-proA/ ZpA963-PKA_C	2.33M Na formate 91mM MES pH 5.5	4°C / 7.5 mg/ml	30% glycerol
ProA 3 repeats	13.5% PEG 1000, 0.1 M Na acetate pH 4.5	23°C / 20 mg/ml	30% ethylene glycol
ProA 4 repeats	43.2 % PEG 1000, 0.1 M Na acetate pH 4.5	23°C / 26 mg/ml	3% PEG 1000
ProA 5 repeats	27.3 % PEG MME 2000, 0.1 M Na acetate pH 4.5	23°C / 50 mg/ml	17.7% PEG MME 2000
ProA-DHR14 2 repeats	33.75% PEG MME 2000 0.1M Tris pH 8.0	23°C / 40 mg/ml	11.25% PEG MME 2000

* The crystal freezing solutions were produced by adding the cryoprotectants to the crystallization solutions.

Supplementary Table 5. Data collection and refinement statistics

Data collection	MrsD-proA	DARPin-proA	PKA_RIIα-proA	PKA_RIIα-DHR14
Space group	$P2_13$	$P2_1$	$P2_1$	C2
Resolution (Å)	2.70 – 50.0	2.60 – 50.0	3.20 – 50.0	2.00 – 50.0
Coordinations				
a, b, c (Å)	167.7, 167.7, 167.7	70.8, 94.7, 70.9	71.6, 135.4, 89.0	179.2 96.5 39.3
a, β, γ (°)	90.0, 90.0, 90.0	90.0, 117.3, 90.0	90.0, 108.8, 90.0	90.0 89.9 90.0
R_{sym}	0.073 (0.554)	0.123 (0.457)	0.109 (0.547)	0.095 (0.402)
I/σI	22.1 (1.5)	9.9 (2.2)	11.2 (1.8)	19.8 (4.2)
Completeness (%)	92.0 (61.0)	99.6 (99.6)	99.4 (99.1)	98.7 (99.5)
Redundancy	3.3 (2.4)	3.4 (3.1)	3.3 (3.0)	3.5 (3.5)
Search probes (PDB code)	1P3Y, 2SPZ	1SVX, 1DEE	2IZX, 2SPZ	2IZX, 5CWH
**Refinement**				
Reflections (work)	39652	25766	26439	44436
Reflections (test)	1998	2003	2003	1977
R_{work}/R_{free}	0.204 / 0.243	0.216 / 0.250	0.239 / 0.285	0.181 / 0.207
No. of protein molecules	4	3	12	2
No. of atoms	6812	4664	8191	3505
Protein	6588	4512	8191	3060
Ligands	212	0	0	0
Water	12	152	0	445
Average B factor (Å ^{2})	105.9	45.9	90.8	29.2
Wilson B factor (Å ^{2})	76.5	31.0	73.1	18.5
R.m.s deviations				
Bond length (Å)	0.006	0.003	0.006	0.009
Bond angles (°)	0.88	0.560	0.990	0.86
Ramachandran plot*				
Favored (%)	96.6	99.5	97.0	100.0
Allowed (%)	3.2	0.5	2.8	0.0
Outliers (%)	0.2	0.0	0.2	0.0
PDB code	5H75	5H76	5H77	5H78

The highest resolution shells are shown in parenthesis.
*Calculated using the program MolProbity [2].

Supplementary Table 6. Data collection and refinement statistics

**Data collection**	**ProA_3 repeats**	**ProA_4 repeats**	**ProA_5 repeats**	**proA-DHR14 2 repeats**
Space group	$P2_1$	P1	$P2_1$	P1
Resolution (Å)	2.70 – 50.0	2.70 – 50.0	3.1 – 50.0	2.70 – 50.0
Coordinations				
a, b, c (Å)	58.7, 46.6, 74.3	55.4 80.8 130.3	80.9 45.6 82.1	48.7, 75.5, 80.0
a, β, γ (°)	90.0 107.3 90.0	89.8 89.6 90.1	90.0 105.7 90.0	84.2, 83.7, 75.2
R_{sym}	0.097 (0.377)	0.085 (0.258)	0.136 (0.555)	0.047 (0.326)
I/σI	15.5 (2.1)	8.2 (2.2)	7.2 (1.0)	15.2 (1.6)
Completeness (%)	98.7 (98.6)	97.0 (94.2)	96.9 (94.2)	95.9 (94.6)
Redundancy	3.3 (3.1)	1.9 (1.8)	2.8 (2.6)	1.8 (1.7)
Search probes (PDB code)	4ZNC, 5COC	4ZNC, 5COC	4ZNC, 5COC	4ZNC, 5CWH
**Refinement**				
Reflections (work)	10490	63216	8287	28731
Reflections (test)	500	2019	420	1437
R_{work}/R_{free}	0.231 / 0.279	0.246 / 0.284	0.270 / 0.314	0.203 / 0.262
No. of protein molecules	2	12	2	2
No. of atoms	2282	18409	3387	6284
Protein	2265	18092	3387	6284
Ligands	0	0	0	0
Water	17	317	0	0
Average B factor ($Å^2$)	94.79	35.14	64.73	86.89
Wilson B factor ($Å^2$)	57.12	29.68	30.90	59.04
R.m.s deviations				
Bond length (Å)	0.004	0.004	0.003	0.004
Bond angles (°)	0.790	0.829	0.650	0.680
Ramachandran plot*				
Favored (%)	96.4	96.5	94.0	98.5
Allowed (%)	3.6	3.5	6.0	1.5
Outliers (%)	0.0	0.0	0.0	0.0
PDB code	5H79	5H7A	5H7B	5H7C

The highest resolution shells are shown in parenthesis.
*Calculated using the program MolProbity [2].

Supplementary Table 7. Data collection and refinement statistics

**Data collection**	**YgjG-proA/ ZpA963-calmodulin**	**YgjG-proA/ ZpA963-PKA_C**	**PKA_RIIα-proA (end-to-end)**
Space group	P1	I222	$C222_1$
Resolution (Å)	2.60 – 50.0	3.40 – 50.0	3.30 – 50.0
Coordinations			
a, b, c (Å)	79.5 155.4 155.5	140.5 153.5 205.7	76.0 174.1 103.6
a, β, γ (°)	84.8 89.7 89.6	90.0 90.0 90.0	90.0 90.0 90.0
R_{sym}	0.059 (0.385)	0.126 (0.880)	0.082 (0.787)
I/σI	14.6 (1.2)	13.7 (1.4)	23.6 (1.6)
Completeness (%)	90.5 (83.4)	99.7 (99.7)	98.9 (100.0)
Redundancy	2.3 (2.0)	6.5 (6.1)	6.3 (5.8)
Search probes (PDB code)	4UOY, 2M5A, 3DVE	4UOY, 2M5A, 4NTS	2IZX, 4ZNC
**Refinement**			
Reflections (work)	210650	31036	10808
Reflections (test)	21070	1493	535
R_{work}/R_{free}	0.204 / 0.240	0.182 / 0.219	0.245 / 0.280
No. of protein molecules	16	2	4
No. of atoms	37132	6616	2553
Protein	37116	6616	2553
Ligands	16	0	0
Water	0	0	0
Average B factor (Å ^{2})	88.02	135.84	165.82
Wilson B factor (Å ^{2})	62.13	102.78	111.56
R.m.s deviations			
Bond length (Å)	0.009	0.010	0.016
Bond angles (°)	1.21	1.53	1.73
Ramachandran plot*			
Favored (%)	95.2	92.3	97.4
Allowed (%)	4.7	7.6	2.6
Outliers (%)	0.1	0.1	0.0
PDB code	5H7D	5X3F	5XBY

The highest resolution shells are shown in parenthesis.
*Calculated using the program MolProbity [2].

Supplementary References

1. Kumar, S. & Bansal, M. Structural and sequence characteristics of long alpha helices in globular proteins. *Biophys J.* **71**, 1574-86 (1996).
2. Chen, V.B. et al. MolProbity: all-atom structure validation for macromolecular crystallography. *Acta Crystallogr. D Biol. Crystallogr.* **66**, 12-21 (2010).
